# Isolation of novel cold-tolerance genes from rhizosphere microorganisms of Antarctic plants by functional metagenomics

**DOI:** 10.3389/fmicb.2022.1026463

**Published:** 2022-11-18

**Authors:** Patricia de Francisco Martínez, Verónica Morgante, José Eduardo González-Pastor

**Affiliations:** ^1^Department of Molecular Evolution, Centro de Astrobiología (CAB), CSIC-INTA, Madrid, Spain; ^2^Centro de Investigación en Recursos Naturales y Sustentabilidad (CIRENYS), Universidad Bernardo O’Higgins, Santiago, Chile

**Keywords:** cold-tolerance, UV-resistance, oxidative stress, functional metagenomics, extreme environments, Antarctic

## Abstract

The microorganisms that thrive in Antarctica, one of the coldest environments on the planet, have developed diverse adaptation mechanisms to survive in these extreme conditions. Through functional metagenomics, in this work, 29 new genes related to cold tolerance have been isolated and characterized from metagenomic libraries of microorganisms from the rhizosphere of two Antarctic plants. Both libraries were hosted in two cold-sensitive strains of *Escherichia coli:* DH10B Δ*csdA* and DH10B Δ*csdA Δrnr*. The *csdA* gene encodes a DEAD-box RNA helicase and *rnr* gene encodes an exoribonuclease, both essential for cold-adaptation. Cold-tolerance tests have been carried out in solid and liquid media at 15°C. Among the cold-tolerance genes identified, 12 encode hypothetical and unknown proteins, and 17 encode a wide variety of different proteins previously related to other well-characterized ones involved in metabolism reactions, transport and membrane processes, or genetic information processes. Most of them have been connected to cold-tolerance mechanisms. Interestingly, 13 genes had no homologs in *E. coli*, thus potentially providing entirely new adaptation strategies for this bacterium. Moreover, ten genes also conferred resistance to UV-B radiation, another extreme condition in Antarctica.

## Introduction

Antarctica is considered, on average, to be the coldest, driest, and windiest continent on Earth and it has the highest average altitude. In fact, its temperatures are really extreme, reaching values between −20 and −60°C in winter, and its average temperature is always below 0°C. Other extreme conditions that limit the development of life in Antarctica are strong winds, periodic freeze–thaw cycles, high sublimation and evaporation rates, long periods of darkness, or high exposures to ultraviolet radiation (Duarte et al., 2018).

Despite these drastic conditions, many organisms have been able to develop different molecular and morphological strategies that allow them to thrive optimally in these cold environments. They are collectively named psychrophiles or cold-adapted organisms (Dhaulaniya et al., 2019). Microorganisms such as bacteria, archaea, algae, fungi, or protists prevail in these cold habitats and have a key role in the transport of energy, nutrient recycling, and mineralization processes, which are vital for the functioning of these terrestrial and aquatic ecosystems (Ruisi et al., 2007). However, drastic low temperatures affect their cell envelope properties and functions by reducing membrane permeability and restricting its flexibility and diffusion rates. Moreover, embedded proteins also decrease their mobility and function, and turgor pressure is normally increased by ice formation or freeze–thaw cycles (Collins and Margesin, 2019). To counteract these deleterious effects, cold-adapted microorganisms usually introduce some changes in their membrane compositions favoring an increase in their fluidity and, therefore, their functionality under low temperatures (Kahlke and Thorvaldsen, 2012). In addition, upregulation of membrane transport proteins has been detected in some psychrophiles, facilitating increased rates of diffusion and transport rates (De Maayer et al., 2014).

Enzymes and proteins are also negatively affected at low temperatures (Dhaulaniya et al., 2019) and their reaction rates might be considerably reduced under these conditions. However, psychrophiles have adapted their enzymes to extreme cold conditions by decreasing their structural (Sindhu et al., 2017) and thermal stability (Maiangwa et al., 2015), favoring its flexibility, specificity, and kinetics by increasing enzyme concentration, and evolving new alternative enzymes whose reaction rate is only controlled by diffusion and is completely independent of temperature (Georlette et al., 2004). In addition, protein and RNA/DNA chaperones play an important role in cold-adaptation counteracting protein misfolding and aggregation as well as stabilizing RNA/DNA secondary structures. In this way, they are responsible for maintaining efficient transcription, translation, and DNA replication rates (Lim et al., 2000).

Cold adaptation is also related to various metabolic adjustments that favor reduced ROS production and the conservation of energy for long-term survival. These metabolic adjustments include the downregulation of primary metabolism pathways, the activation of some alternative secondary pathways, and the metabolism and accumulation of reserve compounds, such as polyhydroxyalkanoate (PHA), which can be used as a dynamic reserve of carbon, nitrogen, and energy, but may also play a key role in cryoprotection, oxidative stress resistance, cell motility, or maintenance of cellular redox balance (Methé et al., 2005; Tribelli et al., 2015; Tribelli and Lopez, 2018).

Furthermore, subzero temperatures normally cause ice formation in the extracellular space and, therefore, osmotic stress, dehydration, cryoinjury, or even cell rupture and death (Fonseca et al., 2016). Cold-adapted microorganisms respond to these deleterious effects by producing different types of cryoprotectants such as compatible solutes (Goordial et al., 2016), ice-binding proteins (antifreeze and ice-nucleating proteins) (Lorv et al., 2014; Voets, 2017), biosurfactants (Kitamoto et al., 2001; Perfumo et al., 2018), or extracellular polymeric substances (Caruso et al., 2018).

All of the cellular cold-adaptation strategies described above have already been well-characterized. Nonetheless, further research effort is needed. The research on psychrophiles is really interesting not only to increase our knowledge about cold-tolerant molecular mechanisms but also to describe new cold-active enzymes with new properties that could confer some advantages for many biotechnological processes (Duarte et al., 2018). Some enzymes from these microorganisms have been used in the food industry to improve milk fermentation and meat and dough quality, in agriculture as biofertilizers, or in textile industry for dye removal or bleaching processes (Gurung et al., 2013; Nigam, 2013). On the other hand, psychrophilic microorganisms have strict requirements for their growth, so reproducing these conditions in the laboratory is quite difficult and many strains are not culturable under standard laboratory conditions. To overcome this problem and with the aim of searching for novel cold-tolerant genes and increasing our knowledge about the molecular mechanisms of cold adaptation, we used a functional metagenomic approach in this work. Through this methodology, we were able to access the entire genetic potential of microorganisms in the rhizosphere of two Antarctic plants: *Deschampsia antarctica* and *Colobanthus quitensis*. This procedure allows a culture-independent analysis of functional genes related to a cold response of microbial genes isolated from the Antarctic samples. As a result, 29 novel cold-tolerant genes were isolated and characterized in this study. Seventeen of them were similar to previously described genes and few of them had already been related to cold-acclimation processes, whereas others had not previously been linked to cold tolerance. Furthermore, 12 hypothetical or unknown proteins were also described in this study. This is a valuable finding since these genes could be related to novel cold-tolerance mechanisms not described until now. All these results offered us a broader view of the cold-adaptation mechanisms that microorganisms have developed under these extreme environments.

## Materials and methods

### Bacterial strains and growth conditions

Three strains of *Escherichia coli* have been used in this study: *E. coli* DH10B (Invitrogen) and two cold-sensitive mutants that we specifically constructed for this screening: *E. coli* DH10B *ΔcsdA* and *E. coli* DH10B *ΔcsdA Δrnr. E. coli* DH10B was used as a host to construct and to maintain the metagenomic libraries, while DH10B *ΔcsdA* and DH10B *ΔcsdA Δrnr* strains were used as hosts for screening for cold-tolerance genes. These three strains were routinely grown in Luria-Bertani (LB) medium (Conda) at 37°C. The growth medium for transformed *E. coli* strains was supplemented with 50 mg/ml ampicillin (Ap) to maintain the pBluescript SKII (+) plasmid. When required, the working concentration of 5-bromo-4-chloro-3-indolyl-β-galactopyranoside (X-Gal) was 40 μg/ml and of isopropyl β-D-1-thiogalactopyranoside (IPTG) was 100 μM.

### Construction of *Escherichia coli* cold-sensitive mutant strains

Two cold-sensitive strains (*E. coli* DH10B *ΔcsdA* and *E. coli* DH10B *ΔcsdA Δrnr*) were constructed from *E. coli* DH10B cells (Invitrogen). The Counter-Selection BAC Modification Kit (Gene Bridges) was used to obtain both cold-sensitive mutants utilizing the Red/ET recombination technique (Gene Bridges) that allows the homologous recombination *in vivo* and, therefore, the specific exchange of our gene of interest (*csdA* or *rnr*) by an antibiotic resistance cassette (kanamycin was selected to obtain the DH10B *ΔcsdA* strain and chloramphenicol for DH10B *ΔcsdA Δrnr*). Specific DNA cassettes were designed and synthetized including the corresponding antibiotic resistance gene, restriction enzymes sites (BamHI and XbaI), and flanking fragments specific for the UTRs regions of *csdA* and *rnr* genes (Integrated DNA Technologies, IDT). *E. coli* DH10B cells were first transformed with the pRedET plasmid (Gene Bridges), which carries the λ phage γ*βα* operon under the control of the arabinose-inducible pBAD promoter and that confers tetracycline resistance. Then, we introduced our insert (previously amplified by PCR (primers are described in Supplementary Table S1) and linearized by BamHI/XbaI digestion) into these cells and homologous recombination was induced by adding 10% arabinose to the medium and incubating for 1 h at 37°C. Selection of positive clones was performed by adding kanamycin (15 μg/ml) or chloramphenicol (20 μg/ml) to the LB-agar media. Correct insertion of the resistance cassette in the bacterial chromosome and the elimination of our gene of interest were verified by PCR (Supplementary Table S1).

### Construction of metagenomic libraries

Two metagenomic libraries have been constructed using DNA isolated from rhizosphere samples of two Antarctic plants: *Colobanthus quitensis* (library C) and *Deschampsia antarctica* (library D). Samples were recovered during the austral summer (February 2011) at Admiralty Bay in King George Island, located in the South Shetland archipelago in the north of Antarctic Peninsula. *Deschampsia* rhizosphere samples were obtained close to the Brazilian Antarctic Station (Estação Antártica Comandante Ferraz; GPS coordinates: 62°03′40″S–58°23′30″W), while the *Colobanthus* samples were collected in the Antarctic Specially Protected Area (ASPA N° 151; GPS coordinates: 62°08′00″S–58°28′10″W) adjacent to the Arctowski Polish Station.

Roots with soil particles tightly adhered to them were collected at a depth of 5 cm and immediately kept in 50 ml tubes containing RNA Later (Sigma) solution. All samples were collected in triplicate and stored frozen at –80°C. In order to extract high-quality and intact metagenomic DNA from the rhizosphere samples, they were thawed and aseptically processed with the BIO101 FastDNA Spin kit for soil (Qbiogene), obtaining 20 μg of DNA per gram of sample. Libraries were constructed using the high-copy number pBluescript II SK (+) vector and the *E. coli* DH10B strain as a host, as previously described (Mirete et al., 2007; González-Pastor and Mirete, 2010). Briefly, the metagenomic DNA was partially digested using Sau3AI and the fragments from 1 to 8 Kbp were isolated by a 0.8% low-melting-point agarose gel electrophoresis with the QIAquick Gel Extraction kit (QIAGEN). The metagenomic DNA fragments were then ligated into the BamHI digested and dephosphorylated pBluescript II SK (+) vector. DNA (100–125 ng) purified from the gel was mixed with the vector in a 1:1 molar ratio. Ligation mixtures were incubated overnight at 16°C using T4 DNA ligase (Roche) and used to transform *E. coli* DH10B cells (Invitrogen) by electroporation with a MicroPulser (Bio-Rad) according to the manufacturer’s instructions (Mirete et al., 2007; González-Pastor and Mirete, 2010). To estimate the average insert size of the library, the plasmids from ten random recombinant clones were isolated on LB-Ap plates and digested using either EcoRI or XbaI and XhoI restriction enzymes (Roche). To amplify these libraries, cells were grown at 37°C for 24 h on LB-agar plates containing ampicillin (approximately 1.3 × 10^4^ cells per plate). Then, cells from each plate were recovered, mixed with LB and 10% glycerol (*w*/*v*), and stored at −80°C.

### Screening for cold-tolerant clones in the metagenomic libraries

Recombinant plasmids from the metagenomic libraries constructed in *E. coli* DH10B cells were extracted using the QIAprep Spin Miniprep kit (Qiagen) and 100 ng of them were used to transform electrocompetent cells of *E. coli* DH10B *ΔcsdA* and DH10B *ΔcsdA Δrnr*, which had been previously prepared according to Dower et al., 1988. To amplify the libraries, after electroporation, the transformed cells were grown in liquid LB-Ap medium at 37°C to increase the number of viable cells around 10^4^ times. To select for cold-tolerant clones, a total of 3 × 10^6^ recombinant clones from each library were spread on LB agar supplemented with Ap, X-Gal, and IPTG (around 10^5^ cells per plate) and incubated at 15°C during 3–5 days until the appearance of colonies. To ensure that the resistance phenotype was not due to the presence of spontaneous chromosomal mutations, resistant colonies were pooled, and their plasmid DNA were isolated and used to transform DH10B *ΔcsdA* or DH10B *ΔcsdA Δrnr* cells. The new retransformed clones were grown again at 15°C on LB agar (Ap, X-Gal, IPTG) medium. Cold-tolerant clones were selected and their isolated recombinant plasmids were digested with XhoI and XbaI (Roche) to identify those which are unique in their restriction patterns.

### *In silico* analysis of cold-tolerant clones

The DNA inserts cloned into pBluescript II SK (+) plasmids isolated from cold-tolerant clones were sequenced on both strands with universal primers M13F/M13R and with other primers specifically designed for primer walking by using the ABI PRISM dye terminator cycle-sequencing ready-reaction kit (PerkinElmer, Waltham, MA, United States) and an ABI PRISM 377 sequencer (PerkinElmer). Sequences were assembled and analyzed with the ApE program. Prediction of potential open reading frames (ORFs) was conducted using the online source ORF Finder, which is available at the NCBI webpage.^1^ The bacterial code was selected by allowing ATG, CTG, GTG, and TTG as alternative start codons for translation to protein sequences. All the predicted ORFs longer than 90 bp were translated and used as queries in BlastP. Their putative function was annotated based on their similarities to protein family domains by using NCBI’s Conserved Domain Database and Pfam (Protein Families).^2^ Those translated ORFs with an E-value higher than 0.001 in the BlastP searches were considered unknown proteins. For DNA-binding domain prediction, two different programs were used with default settings: DPP-PseAAC (Rahman et al., 2018)^3^ and DNABIND (Liu and Hu, 2013).^4^

### Cold-tolerance tests

All the positive clones isolated from the cold screening were tested in liquid and solid media at 15°C. Two controls were introduced in each experiment: a positive control (DH10B strain transformed with the empty pBluescript II SK (+) vector) and a negative control (DH10B *ΔcsdA* or DH10B *ΔcsdA Δrnr* strains transformed with the empty pBluescript II SK (+) vector). Solid medium tests were carried out by drop assay using cell cultures grown overnight at 37°C in LB-Ap liquid medium and whose OD_600nm_ values were adjusted to 1. The following serial dilutions were made: 1, 1/5, 1/10, 1/50, 1/100, and 1/1,000 and they were distributed as drops of 10 μl on LB-Ap solid medium. Plates were incubated at 15°C for 10 days. The results were confirmed by repeating these tests at least three times using independent cultures. We also checked that all the strains had initially been adjusted to a similar cell density and that all of them have a similar viability by carrying out the same drop assay at 37°C (without a cold stress). In this control assay, we made the following serial dilutions: 1, 10^−1^, 10^−2^, 10^−3^, 10^−4^, and 10^−5^, to observe possible cell viability differences between cold-tolerant clones and control strains.

Liquid medium tests were used to confirm the cold-tolerance results obtained in solid medium tests. They were performed in 96-well plates with cell cultures whose OD_600nm_ values had previously been adjusted to 0.03. Cells were cultured in LB-Ap liquid medium at 15°C and 80 rpm for 7 days. Growth curves were constructed by measuring OD_600nm_ values four times per day using the microplate reader SPECTROstar Nano (BMG Labtech). Non-inoculated wells served as blanks and their values were subtracted from those obtained in inoculated wells. Growth curves were built using the DMFit online tool included in the Combase website,^5^ according to Baranyi and Roberts model (Baranyi and Roberts, 1994). After obtaining the growth curves, we calculated two kinetic parameters for all of them: the specific growth rate (*μ*, h^−1^) and the generation time (Tg, h). The μ was defined as the slope of the exponential phase of the growth curve (linear phase) and the Tg was calculated using the following formula: Tg = (Ln 2)/*μ*. Liquid medium tests were repeated at least three times using independent cultures to confirm the results and six replicates of each strain were introduced in each plate. Moreover, growth curves were also built at 30°C to confirm that all the strains were able to grow at the same rate independently of the metagenomic insert that they contain. These control curves were obtained by measuring OD_600nm_ values every hour for 30 or 40 h in the TECAN Infinite M Nano microplate reader.

### Subcloning of independent genes to identify those that confer cold tolerance

To determine which genes could be directly involved in cold resistance in those recombinant plasmids containing more than one gene, all of them were cloned individually in the pBluescript SKII (+) vector. Therefore, fragments containing these genes and a region of ∼ 200 bp located upstream of the start codon, which probably incorporates native expression sequences (promoters and ribosome binding sites), were amplified by PCR using M13 primers and specific primers (Supplementary Table S1). The following reaction mixture was used in each PCR assay: 100 ng of plasmid DNA, 500 μM of each of the four dNTPs, 2.5 U of *Pfu* Turbo DNA Polymerase (Agilent), and 200 nM of each forward and reverse primers up to a total volume of 50 μl. The PCR amplification program used was as follows: 1 cycle of 5 min at 95°C, 30 cycles of 45 s at 95°C, 30 s at 52°C and 10 min at 72°C, and finally, 1 cycle of 10 min at 72°C. PCR amplification products were excised from agarose gels and purified using the QIAquick Gel Extraction kit (Qiagen). Purified PCR products were digested with the appropriate restriction enzymes (XbaI/XhoI, XbaI/KpnI, or XbaI/SalI, Roche) and ligated into pBluescript II SK (+; using a 1:6 ratio) previously digested with the same restriction enzymes. DH10B *ΔcsdA* or DH10B *ΔcsdA Δrnr* cells were transformed with these recombinant plasmids and their response was compared to the original clones carrying complete environmental DNA fragments by cold tests in solid and liquid media. All genes were subcloned in the same orientation as the original clone.

### Overexpression of some *Escherichia coli* homologs in the cold-sensitive mutant strains

The *E. coli* homologs for some of the cold-tolerant genes isolated in this work were overexpressed in the *E. coli* DH10B *ΔcsdA* strain. These genes were selected from the genome of the *E. coli* DH10B strain by Blast (NCBI) and only those that shared a considerable similarity with the cold-tolerance genes (%ID > 25% and *E*-values < 0.001) were overexpressed. These *E. coli* genes were amplified by PCR using the genomic DNA of the DH10B strain as a template (the primers are described in Supplementary Table S1) and similarly subcloned into pBluescript II SK (+) vector as described in the previous section*. E. coli* DNA was isolated using the Wizard Genomic DNA Purification Kit as recommended by the manufacturer (Promega). *E. coli* DH10B *ΔcsdA* cells were transformed with these recombinant plasmids and the resulting strains overexpressing cold-tolerant homologs were tested for cold tolerance at 15°C in solid and liquid media as described above.

### Ultraviolet-radiation resistance test by drop assay

In order to investigate whether the cold-tolerant clones may be resistant to other types of stress conditions, they were exposed to UV-B radiation. First, overnight cultures of these clones in LB-Ap liquid medium were adjusted to an OD_600 nm_ of 1 and serial dilutions were made (1/2, 1/4, 1/8, 1/10, and 1/100). Then, a 10 μl drop of each dilution was inoculated on LB-Ap solid medium. As negative controls, the cold-sensitive strains DH10B *ΔcsdA* and DH10B *ΔcsdA Δrnr*, carrying the empty pBluescript II SK (+) vector, were used. Cells were irradiated with UV-B radiation at a dose of 4 mJ/cm^2^ and incubated overnight at 37°C. Then, their UV-resistance was evaluated by comparing the growth of cold-tolerant clones with negative controls. A control assay was performed with the same strains but they were not irradiated to verify their similar viability regardless of the applied treatment. The assay was repeated under the same conditions for the clones most resistant to UV-B radiation and their respective subclones were also tested to identify which genes were responsible for the UV-resistance observed in the complete clones.

## Results

### Screening for genes involved in cold tolerance from the rhizosphere of Antarctic plants

**Figure 1:**
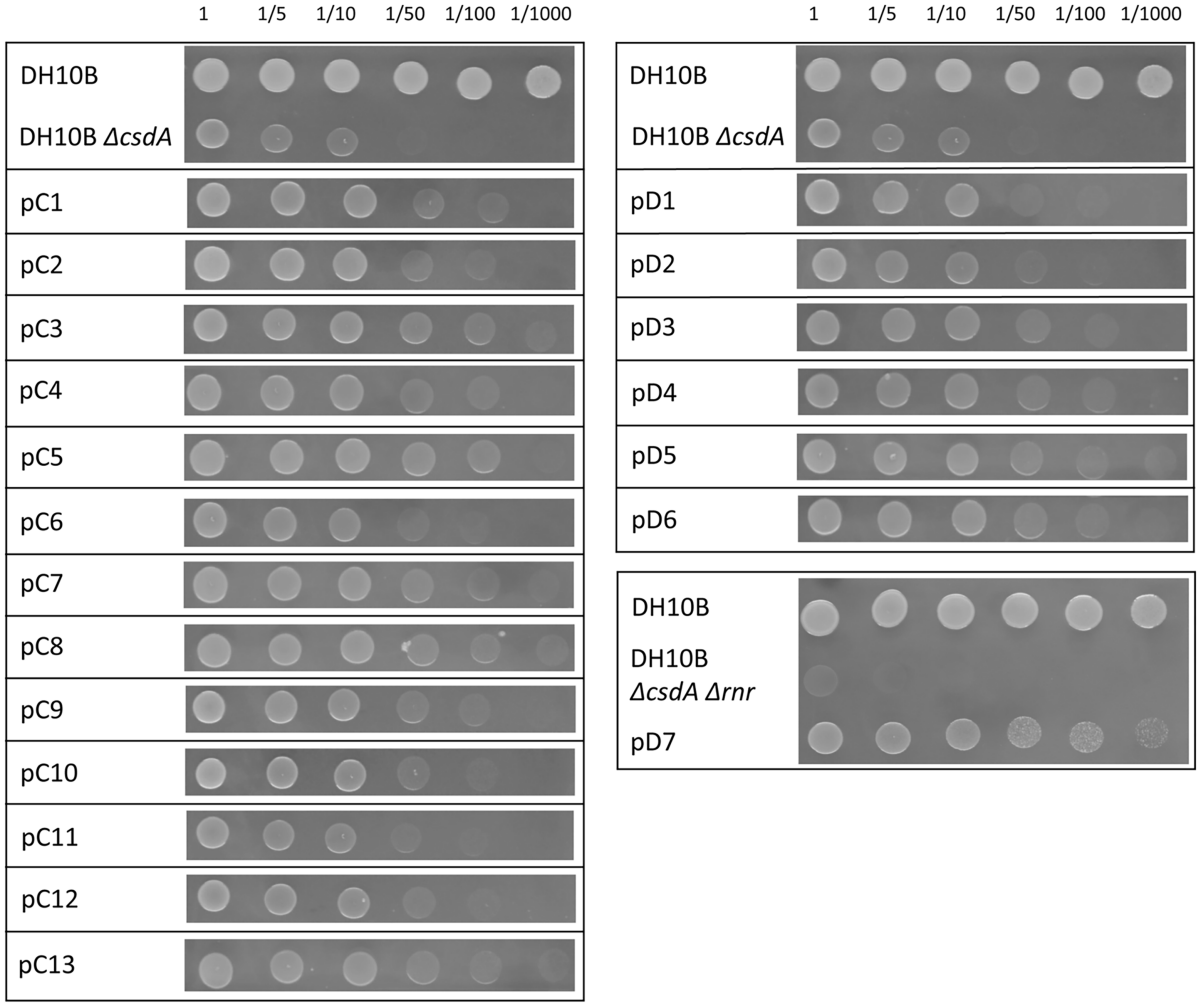
Drop assay of the 20 cold-resistant clones isolated from Antarctic metagenomic libraries: library C constructed from *Colobanthus quitensis* rhizosphere samples and library D from *Deschampsia antarctica* rhizosphere samples. The DH10B strain carrying an empty pBluescript vector was used as a positive control and both cold-sensitive strains (DH10B Δ*csdA* and DH10B Δ*csdA* Δ*rnr*) also carrying empty pBluescript vectors were used as negative controls. The OD_600 nm_ of overnight cultures was adjusted to values of 1.0 and serial dilutions were performed. 10 μl drops were inoculated on LB-Ap_50_ plates and grown at 15°C for 10 days. Each experiment was repeated at least three times using independent cultures.

**Figure 2:**
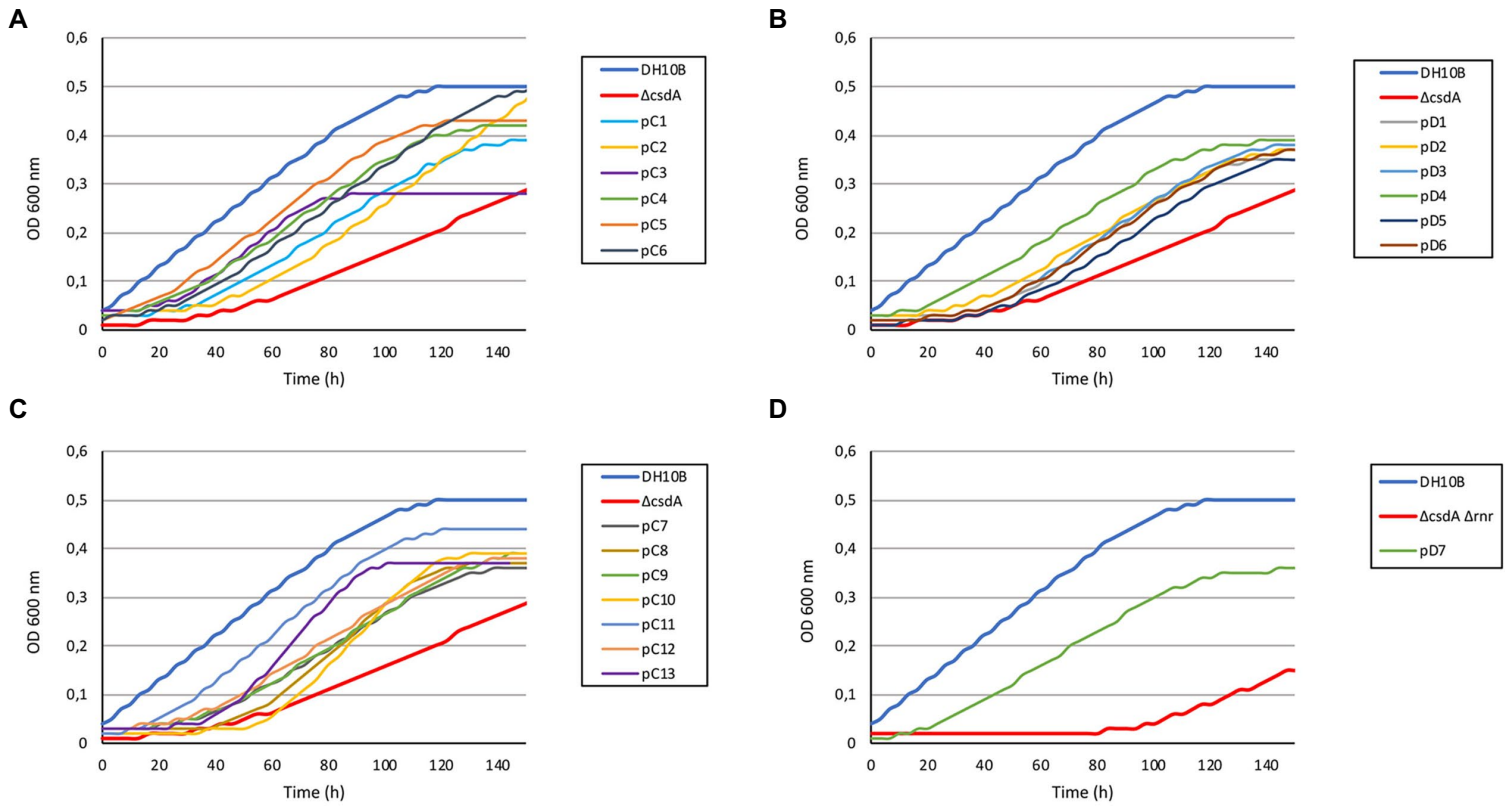
Growth curves of the 20 cold-resistant clones isolated from Antarctic metagenomic libraries. The DH10B strain carrying an empty pBluescript vector was used as a positive control and both cold-sensitive strains (DH10B Δ*csdA* and DH10B Δ*csdA* Δ*rnr*) also carrying empty pBluescript vectors were used as negative controls. All the clones were grown during 5 h at 37°C with slight agitation and then their OD_600 nm_ values were adjusted to 0.03. They were grown in 96-well microtiter plates during 140 h at 15°C with a slight agitation (80 rpm) and OD measures were taken in the SPECTROstar Nano (BMG Labtech) four times per day. Six replicates per clone were introduced in each assay and each experiment was repeated at least three times using independent cultures to corroborate the results. **(A)** Results obtained for 6 cold-resistant clones (pC1–pC6) isolated from library C (*Colobanthus quitensis*) in the screening performed with the single cold-sensitive mutant (DH10B Δ*csdA*). **(B)** Results obtained from the remaining 7 cold-resistant clones isolated from library C (pC7–pC13) in the screening performed with the single cold-sensitive mutant. **(C)** Results obtained for the 6 cold-resistant clones (pD1–pD6) isolated from library D (*Deschampsia antarctica*) in the screening performed with the single cold-sensitive mutant. **(D)** Results obtained for the cold-resistant clone pD7 isolated from library D in the screening performed with the cold-sensitive double mutant (DH10B Δ*csdA* Δ*rnr*).

In order to search for genes that could confer cold tolerance to *E. coli* from microorganisms of the rhizosphere of Antarctic plants, two metagenomic libraries were constructed using metagenomic DNA from rhizosphere samples of the *Colobanthus quitensis* (library C) and *Deschampsia antarctica* (library D) plants. Approximately 1,100,000 recombinant clones were obtained from each library. The average size of the DNA fragments cloned in the pBluescript II SK (+) vector used for the libraries was 4.5 kb. Each library was amplified as described in Experimental Procedures. The DH10B strain used as a host for these libraries can grow at low temperatures, and was therefore not suitable for cold screening of metagenomic libraries. To avoid this problem, two cold-sensitive strains were constructed, DH10B *ΔcsdA* and DH10B *ΔcsdA Δrnr*, and the metagenomic libraries (C and D) were transferred to these strains. The *csdA* gene encodes a DEAD-box RNA helicase that is essential for the cold-acclimation process (Phadtare, 2011) and the *rnr* gene encodes an exoribonuclease that is also induced at low temperatures. A single mutant for the *rnr* gene is not cold sensitive but the double mutant *ΔcsdA Δrnr* grows more slowly at 30°C and cannot grow at all at moderate low temperatures (20°C) (Awano et al., 2007; Phadtare, 2011). The growth of these two mutant strains was tested at 15°C in solid and liquid medium, and it was observed that they started to grow later (higher lag phases) and slower (longer generation times) than the parental strain DH10B (Figures 1, 2; Supplementary Table S2). Therefore, to detect genes conferring cold tolerance, the metagenomic libraries in DH10B *ΔcsdA* and DH10B *ΔcsdA Δrnr* strains were grown on solid LB-Ap-medium at 15°C for a variable period of time between 3 and 5 days, conditions in which the same strains harboring empty pBluescript II SK (+) plasmids did not grow.

A total of 60 cold-tolerant colonies (32 from library C and 28 from library D) were obtained from both libraries hosted in the cold-sensitive strain DH10B *ΔcsdA*. To ensure that the resistance phenotype was not due to the presence of spontaneous mutations, all these colonies were pooled and their recombinant plasmids were isolated and used to transform DH10B *ΔcsdA* cells. Finally, after re-isolating under cold conditions and studying the restriction patterns (obtained with XhoI and XbaI restriction enzymes) of 40 colonies from each library, we found 13 different clones from library C (pC1–pC13) and 6 clones from library D (pD1–pD6) that showed a clear cold-tolerance phenotype (Figure 1). On the other hand, in the screening performed with the double mutant DH10B *ΔcsdA Δrnr*, which is more sensitive to low temperatures than DH10B *ΔcsdA*, only one cold-tolerant colony was obtained from library D (pD7) after retransforming and analyzing 30 colonies from each library, following the same protocol described for the screening performed in the single mutant DH10B *∆csdA* (Figure 1).

The DH10B *ΔcsdA* and DH10B *ΔcsdA Δrnr* strains transformed with the recombinant plasmids also showed a better growth rate under low temperatures (15°C) in liquid media (Figure 2) than the cold-sensitive strains carrying an empty vector. Growth parameters calculated from these tests are compiled in Supplementary Table S2, showing that cold-tolerant clones were able to grow faster (lower generation time) or start to grow earlier than cold-sensitive mutants. On the other hand, no differences in growth rates were observed when cells were grown at 37°C on solid media (see Supplementary Figure S1) or when they were grown at 30°C in liquid media (Supplementary Figure S2). These results supported the idea that the environmental genes cloned in the pBluescript II SK (+) vector specifically contributed to an improvement of *E. coli* growth at low temperatures but not to a general cellular development as differences were not detected under optimal (37°C) or suboptimal (30°C) conditions.

### Identification of genes conferring cold tolerance

**Figure 3:**
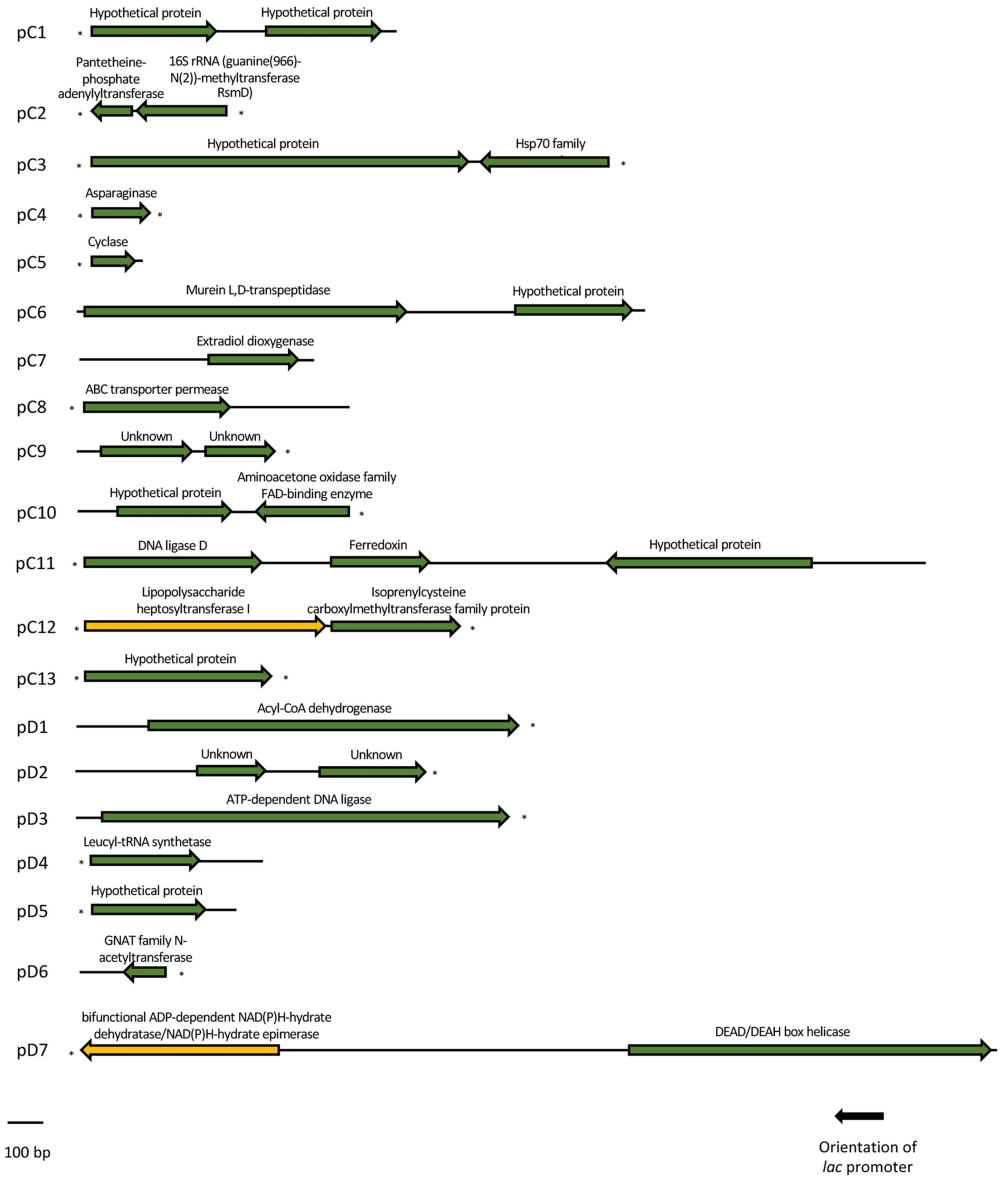
Schematic organization of the ORFs identified in each of the 20 cold-resistant clones. Arrows indicate the location and the transcriptional orientation of all the putative genes, being marked in green the ones related to a cold-tolerant phenotype and in yellow the ones that were not related to cold-tolerance. Truncated ORFs are indicated with an asterisk.

The metagenomic DNA fragments of each plasmid were sequenced by prime walking and a total of 29 genes were predicted in the 13 clones (pC1–pC13) isolated from library C and in the 6 clones (pD1-pD6) from library D in the screening performed with the DH10B *ΔcsdA* strain (Table 1; Figure 3). Moreover, two genes were predicted in the cold-tolerant pD7 clone isolated in the screening performed with the double mutant (Table 1; Figure 3). Sequence analyses of these environmental DNA fragments revealed the presence of a single open reading frame (ORF) in pC4, pC5, pC7, pC8, pC13, pD1, pD3, pD4, pD5, and pD6; two ORFs in pC1, pC2, pC3, pC6, pC9, pC10, pC12, pD2, and pD7; and three ORFs in pC11. The G + C content of these DNA fragments ranged from 39.2 to 76.1%, indicating their diverse phylogenetic origin. Most of the genes analyzed in this study encode amino acid sequences similar to bacterial proteins. Using the BLASTp tool (NCBI website), we tried to relate them with previously characterized proteins, but 8 of them were similar to conserved hypothetical proteins (28%) and 4 were classified as unknown proteins (14%) as no similarity was found between them and previously annotated proteins (Table 1; Figure 3). Using the NCBI Conserved Domain Database (CDD) and the Pfam Database to functionally categorize the recovered genes, a large variety of domains could be predicted (Table 1). In addition, DNA-binding domains were predicted in some sequences using two bioinformatics programs (Table 1).

**Table 1:** Description of cold-resistance clones isolated from metagenomic libraries C and D using DH10B *csdA* and DH10B Δ*csdA* Δ*rnr* strains as hosts, similarities with other known sequences and conserved domains found in them.

ID clone	GenBank accession number	Insert length (bp)	% GC	ORF^a^	Truncated ORFs	Length (aa)^b^	Closest similar protein (microorganism; accession no.)^c^	Identity (*E* value)	Length (aa)	Conserved domains (Conserved Domain Database (NCBI) and Pfam)	DNA-binding domain prediction^d^
pC1	MZ835316	1,179	56.8	(1)	N-term	160	Hypothetical protein (*Acidobacteria bacterium*; OLD28119.1)	76% (2*E*–85)	275	***−***	++
				(2)	No	147	Hypothetical protein (*Acidobacteria bacterium*; PYQ34911.1)	41% (1*E*–19)	144	***−***	None
pC2	MZ835317	514	76.1	(1)	C-term	49	Pantetheine-phosphate adenylyltransferase (*Actinocorallia hervida*; WP 123662510.1)	86% (2*E*–20)	161	Nucleotidyltransferase superfamily Phosphopantetheine adenylyltransferase Cytidylyltransferase-like	None
				(2)	N-term	113	16S rRNA (guanine(966)-N(2))-methyltransferase RsmD (*Sphaerisporangium sp*; WP 113983382.1)	65% (4*E*–36)	190	S-adenosyl methionine-dependent methyltransferase 16S rRNA (guanine(966)-N(2))-methyltransferase RsmD	None
pC3	MZ835318	1,977	64.1	(1)	N-term	479	Hypothetical protein (*Candidatus Melainabacteria bacterium*; OGI06829.1.1)	46% (2*E*–137)	730	S-adenosyl methionine-dependent methyltransferase Spermidine synthase	+
				(2)	N-term	162	Hsp70 family protein (*Acidobacteria bacterium*; RPI53355.1)	73% (2*E*–76)	439	Hsp70 protein Nucleotide-binding domain of the sugar kinase/HSP70/actin superfamily	+
pC4	MZ835319	219	54.8	(1)	N-term and C-term	73	Asparaginase (*Burkholderia plantarii*; WP 042628756.1)	92% (8*E*–38)	382	Bacterial L-asparaginases and related enzymes	None
pC5	MZ835320	194	61.9	(1)	N-term	54	Cyclase (*Rhodopseudomonas palustris*, WP 044410308.1)	80% (9*E*–25)	153	−	++
pC6	MZ835321	2,169	67.8	(1)	No	409	Murein L,D-transpeptidase (*Sorangium cellulosum*, WP 020741004.1)	50% (2*E*–114)	453	L-D-transpeptidase catalytic domain (YkuD)	None	
						(2)	No	148	Hypothetical protein (*Deltaproteobacteria bacterium*, RLB59251.1)	35% (2*E*–19)	159	−	++
pC7	MZ835322	897	63.8	(1)	No	113	Extradiol dioxygenase (*Candidatus Rokubacteria bacterium*; PYO01824.1)	76% (5*E*–59)	118	−	None
pC8	MZ835323	2,130	60.3	(1)	N-term	556	ABC transporter permease (*Chloroflexi bacterium*, RLC95438.1)	39% (2*E*–102)	986	FtsX-like permease family	None
pC9	MZ835324	754	64.5	(1)	No	115	Unknown	−	−	−	++
				(2)	C-term	87	Unknown	−	−	−	None
pC10	MZ835325	1,907	54	(1)	No	435	Hypothetical protein (*Acidobacteria bacterium*; PYS51009.1)	73% (0.00)	436	Putative ATP-dependent DNA helicase Putative DNA-binding domain	++
				(2)	N-term	117	Aminoacetone oxidase family FAD-binding enzyme (*Blastocatellia bacterium*; PWT92013.1)	65% (9*E*–47)	392	HI0933-like protein	None
pC11	MZ835326	3,214	65.2	(1)	N-term	223	DNA ligase D (*Acidobacteria bacterium*; PYR88131.1)	83% (6E-125)	718	ATP-dependent DNA ligase	+
				(2)	No	124	Ferredoxin (*Acidobacteria bacterium*; PYR75981.1)	74% (5*E*–62)	122	Thioredoxin (TRX)-like [2Fe-2S] Ferredoxin (Fd) family	+
				(3)	No	315	Hypothetical protein (*Acidobacteria bacterium*; PYQ53204.1)	36% (7*E*–33)	256	−	+
pC12	MZ835327	1,420	58.5	1	N-term	305	Lipopolysaccharide heptosyltransferase I (*Acidobacteria bacterium*; PYS36748.1)	78% (1*E*–174)	362	Glycosyltransferase GTB type superfamily ADP-heptose: LPS heptosyltransferase	++
				(2)	C-term	164	Isoprenylcysteine carboxyl methyltransferase family protein (*Acidobacteria bacterium*; PYS59682.1)	76% (3*E*–86)	177	Phospholipid methyltransferase	+
pC13	MZ835328	711	64	(1)	N-term and C-term	237	Hypothetical protein (*Verrucomicrobia bacterium*; PYJ85122.1)	70% (2*E*–114)	357	Alpha/beta hydrolase Enol-[acyl-carrier-protein] reductase (NADH) Serine aminopeptidase	+
pD1	MZ835329	1,689	60	(1)	C-term	471	Acyl-CoA-dehydrogenase (*Xanthomonadaceae bacterium*; ODU34393.1)	86% (0.00)	595	Acyl-CoA dehydrogenase	None
pD2	MZ835330	1,337	50.7	(1)	No	86	Unknown	−	−	−	+
				(2)	C-term	133	Unknown	−	−	−	None
pD3	MZ835331	1,654	54.4	(1)	C-term	518	ATP-dependent DNA ligase (*Acidobacteria bacterium*; PYX11037.1)	72% (0.00)	567	ATP-dependent DNA ligase domain	++
pD4	MZ835332	659	57.8	(1)	N-term	134	Leucyl-tRNA synthetase (*Kouleothrix aurantiaca*; KPV53383.3)	81% (1*E*–64)	920	tRNA synthetases class I Anticodon binding domain of tRNA	None
pD5	MZ835333	551	39.2	(1)	N-term	143	Hypothetical protein (*Candidatus Levybacteria bacterium*; OGH24733.1)	54% (3*E*–43)	504	−	None
pD6	MZ835334	335	67.8	(1)	N-term	52	GNAT family N-acetyltransferase (*Kribbella sp*; WP_077019138.1)	46% (1*E*–08)	243	−	None
pD7^e^	MZ835335	3,502	57.8	1	C-term	251	Bifunctional ADP-dependent NAD(P)H-hydrate dehydratase/NAD(P)H-hydrate epimerase (*Chthoniobacter flavus*; WP_006981019.1	54.79% (5*E*–74)	499	YjeF-related protein N-terminus Putative carbohydrate kinase Ribokinase/pfkB superfamily	+
				(2)	No	460	DEAD/DEAH box helicase (*Nitrosospira sp.,* WP_090907573.1)	90.93% (0.00)	463	N-terminal helicase domain of the DEAD-box helicase superfamily Superfamily II DNA and RNA helicase ATP-dependent DNA helicase RecQ ATP-dependent RNA helicase SrmB	+

a ORFs involved in cold resistance are indicated in parentheses.

b aa, amino acids.

c Those sequences with an *E*-value higher than 0.001 in BLASTP searches were considered to be unknown proteins.

d Each (+) sign means a positive prediction with one of the two bioinformatics programs (DPP-PseAAC and DNABIND) used to predict DNA-binding domains described in “Materials and methods.”

e Clone isolated in the second screening performed with the double mutant *DH10B ΔcsdA Δrnr*.

The recombinant plasmids pC4, pC5, pC7, pC8, pC13, pD1, pD3, pD4, pD5, and pD6 contained only one ORF each, which are responsible for their cold-tolerance phenotype (Table 1; Figures 1, 2). These clones encode diverse proteins, most of them previously annotated in different organisms. Briefly, pC4 encodes an asparaginase, pC5 a cyclase, pC7 an extradiol dioxygenase, pC8 an ABC transporter permease, pC13 a hypothetical protein, pD1 an acyl-CoA-dehydrogenase, pD3 an ATP-dependent DNA ligase, pD4 a leucyl-tRNA synthetase, pD5 a hypothetical protein, and pD6 a GNAT family N-acetyltransferase (Table 1). However, the rest of the clones contained recombinant plasmids with two or more ORFs that were independently subcloned to define the putative genes responsible for cold tolerance. The results from solid and liquid tests comparing the tolerance of each subclone to the complete clone and to control strains are compiled in Supplementary Figures S3, S4. The DNA insert of pC1 contains two ORFs, both encoding hypothetical proteins. Both genes showed cold tolerance when they were subcloned independently and were able to grow similarly to the complete clone pC1 at 15°C in solid and liquid media (Supplementary Figures S3, S4A). In the case of the DNA insert from pC2, two ORFs were identified: *orf1* encoding a pantetheine-phosphate adenylyltransferase and *orf2* encoding a 16S rRNA methyltransferase. The clones harboring each one of these ORFs were cold resistant compared to our negative control and they were able to grow faster at 15°C in solid and liquid media than the DH10B *ΔcsdA* strain and even the complete clone (Supplementary Figures S3, S4B). The DNA insert of pC3 contains two ORFs: *orf1* encodes a hypothetical protein and *orf2* encodes a Hsp70 family protein. Both ORFs clearly conferred tolerance to low temperatures but *orf2* is slightly more resistant than *orf1* when subcloned independently (Supplementary Figures S3, S4C). In the case of the DNA insert of pC6, two ORFs were identified, encoding a murein L,D-transpeptidase and a hypothetical protein, respectively. Both genes showed cold tolerance when they were subcloned independently (Supplementary Figures S3, S4D). The DNA insert of pC9 also contains two ORFs, both encoding unknown proteins. Both ORFs conferred cold tolerance when independently subcloned (Supplementary Figures S3, S4E). pC10 clone contained a recombinant insert with two possible ORFs, encoding a hypothetical protein and an aminoacetone oxidase, respectively. Both ORFs showed a similar response to low temperatures when independently subcloned and this response was also similar to that observed for the complete clone, thus both subclones were related to cold tolerance (Supplementary Figures S3, S4F). Three ORFs have been described in the DNA insert of pC11: *orf1* encoding a DNA ligase, *orf2* encoding a ferredoxin and *orf3* encoding a hypothetical protein. These three ORFs showed a similar response to cold when they were subcloned independently and it was very similar to the cold tolerance showed by the complete clone (Supplementary Figures S3, S4G). pC12 clone contained a recombinant insert with two ORFs: *orf1* encodes a lipopolysaccharide heptosyltransferase and *orf2* encodes an isoprenylcysteine carboxyl methyltransferase. In this case, *orf2* seemed to be the main responsible for the cold tolerance showed by pC12, as *orf1* grew considerably slower than *orf2* at 15°C (Supplementary Figure S4H). This difference was best detected in the liquid test. The DNA insert of pD2 also contains two ORFs, both encoding unknown proteins. Both ORFs seemed to be responsible for cold tolerance (Supplementary Figures S3, S4I). Finally, pD7 clone isolated from the double mutant screening was also formed by two ORFs: *orf1* encodes a bifunctional ADP-dependent NAD(P)H-hydrate dehydratase/NAD(P)H-hydrate epimerase and *orf2* encodes a DEAD/DEAH box helicase. The second one was clearly related to cold tolerance (Supplementary Figures S3, S4J), whereas the *orf1* did not confer cold tolerance when independently subcloned.

### Evaluation of cold tolerance conferred by overexpressed *Escherichia coli* homologs in the cold-sensitive mutant DH10B *ΔcsdA*

**Figure 4:**
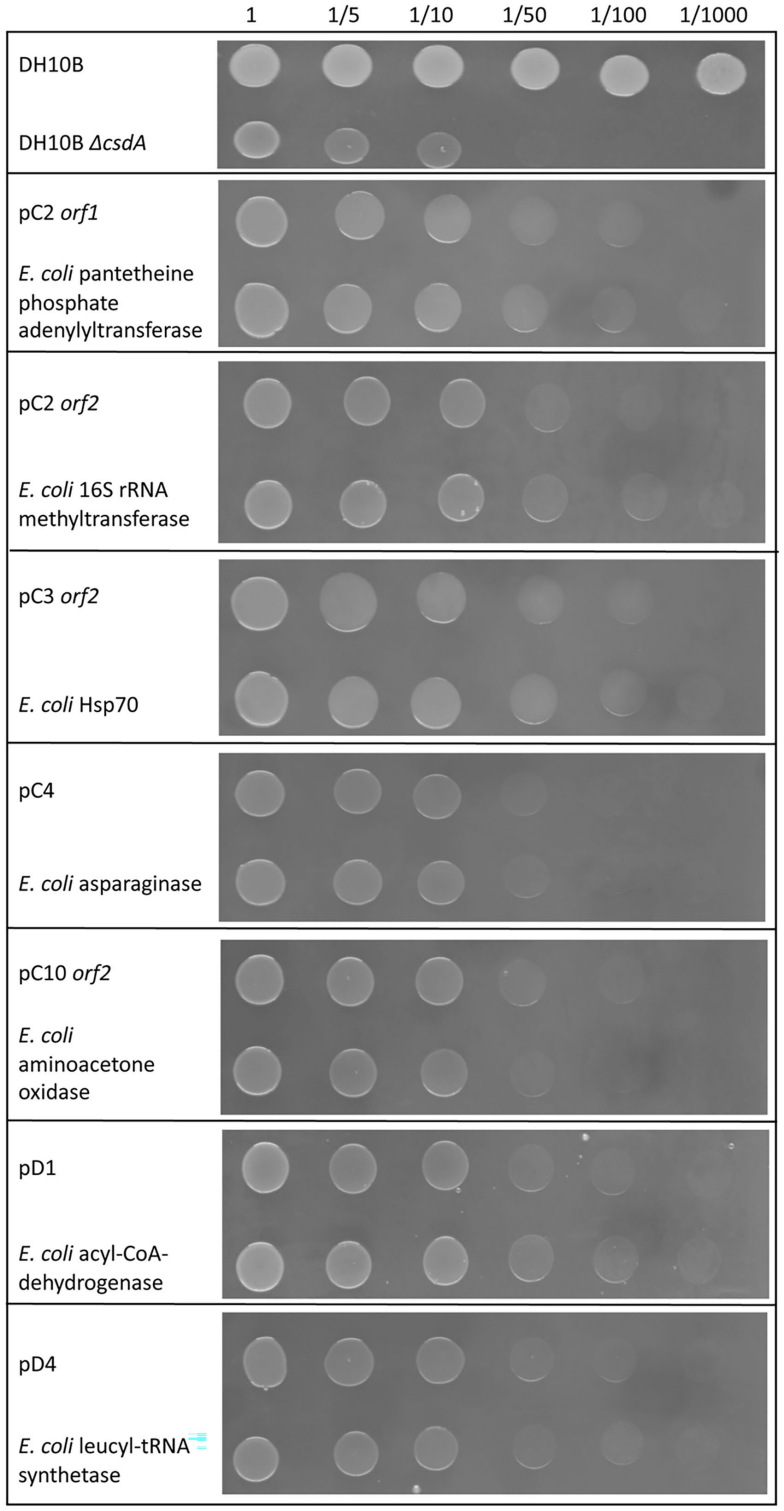
Comparative drop assay performed with some of the cold-tolerant genes characterized in this study and their homologous genes in *Escherichia coli* (see Table 2) expressed in the DH10B Δ*csdA* strain. DH10B strain carrying an empty pBluescript vector was used as a positive control and the single cold-sensitive mutant (DH10B Δ*csdA*) also carrying an empty pBluescript vector was used as a negative control. The cell density of overnight cultures was adjusted to OD_600 nm_ values of 1.0, serial dilutions were performed and 10 μl drops of each dilution were inoculated on LB-Ap_50_ plates and grown at 15°C for 10 days. Each experiment was repeated at least three times using independent cultures.

**Figure 5:**
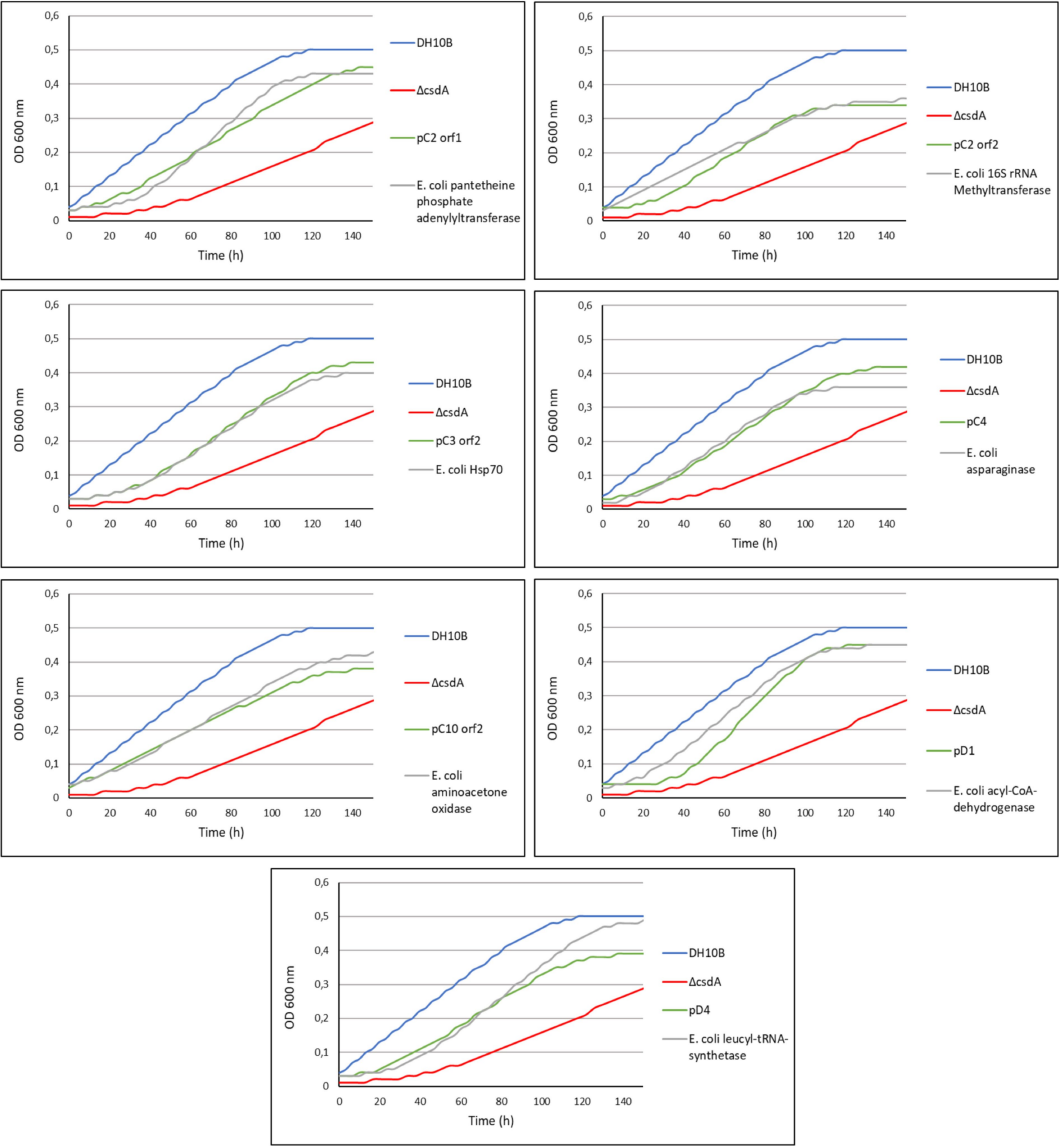
Comparison of the growth curves of some of the cold-tolerant genes characterized in this study and their homologous genes in *E. coli*. DH10B strain carrying an empty pBluescript vector was used as a positive control and the single cold-sensitive mutant (DH10B Δ*csdA*) also carrying an empty pBluescript vector was used as a negative control. All clones were grown during 5 h at 37°C with a slight agitation and then their OD_600 nm_ values were adjusted to 0.03. They were grown in 96-well microtiter plates during 140 h at 15°C with a slight agitation (80 rpm) and OD measures were taken in the SPECTROstar Nano (BMG Labtech) four times per day. Six replicates per clone were introduced in each assay and each experiment was repeated at least three times using independent cultures to corroborate the results.

We found *E. coli* homologs for 16 of the 29 cold-tolerant genes described in this work (55% of the genes; Table 2). For the rest of the genes (45% of the genes), we did not find any homologs in the *E. coli* genome, so they suppose totally new functions for our cold-sensitive strain. Of the 16 *E. coli* homologs, only 10 were identified in the genome of the DH10B strain. Seven of them were overexpressed in the DH10B *ΔcsdA* strain and their cold tolerance was tested in solid and liquid medium at 15°C in comparison with their respective genes identified in this study (Figures 4, 5). We excluded from these overexpression tests the *E. coli* homologs for the pC10 *orf1* and pC13 genes, since they encode hypothetical proteins, and also for pD7 *orf2*, which encodes an RNA helicase that could be complementing the *csdA* mutation in the cold-sensitive *E. coli* strain (Table 1). The overexpression of the seven *E. coli* homologs also increased cold tolerance and these transformed clones were able to grow similarly to the cold-resistant clones originally isolated from the Antarctic libraries (Figures 4, 5). These results suggest that the proteins encoded by these *E. coli* genes might be directly or indirectly involved in cold-adaptation processes of this bacterium and, therefore, the overexpression of their environmental homologs also provided an advantage to grow at low temperatures.

**Table 2:** *Escherichia coli* proteins homologous to cold-tolerant proteins encoded by the genes isolated from Antarctic metagenomic libraries.

Clone name	Homologous *E. coli* protein [Accession number]	% ID; *E*-value
pC2 *orf1*	Pantetheine-phosphate adenylyltransferase [ACB04684.1]^*^,^a^	44% ID; 5*E*–18
pC2 *orf2*	16S rRNA (guanine(966)-N(2))-methyltransferase [ACB04521.1]^*^,^a^	34% ID; 4*E*–12
pC3 *orf2*	Predicted chaperone [ACB03241.1]^*^,^a^	27% ID; 3*E*–11
pC4	L-asparaginase 2 (ansB) [WP_000394140.1]^*^,^a^	59% ID; 1*E*–19
pC10 *orf1*	ATP-dependent DNA helicase RecG [MHO04344.1]^*^	26% ID; 3*E*–21
pC10 *orf2*	Aminoacetone oxidase family FAD-binding enzyme [ACB04545.1]^*^,^a^	50% ID; 5*E*–35
pC11 *orf1*	DNA ligase D [MRF42153.1]	27% ID; 6*E*–12
pC11 *orf2*	(2Fe-2S) ferredoxin domain-containing protein [TFQ09129.1]	36% ID; 3*E*–10
pC12 *orf1*	Lipopolysaccharide heptosyltransferase II [MSF00387.1]	28% ID; 3*E*–21
pC12 *orf2*	Isoprenylcysteine carboxyl methyltransferase family protein [EFD8170757.1]	38% ID; 3*E*–11
pC13	Alpha/beta hydrolase [WP_113373543.1]^*^	31% ID; 5*E*–05
pD1	Acyl-CoA-dehydrogenase [ACB02896.1]^*^,^a^	26% ID; 8*E*–16
pD3	ATP-dependent DNA ligase [MRF43127.1]	30% ID; 2*E*–41
pD4	Leucyl-tRNA synthetase [ACB01864]^*^,^a^	40% ID; 1*E*–27
pD7 *orf1*	Bifunctional ADP-dependent NAD(P)H-hydrate dehydratase/NAD(P)H-hydrate epimerase [WP_130933370.1]	37% ID; 3*E*–25
pD7 *orf2*	RNA helicase [ACB01998.1]^*^	47% ID; 2*E*–110

^*^Homologs identified in *E. coli* DH10B strain. ^a^*E. coli* DH10B homolog genes selected for overexpression experiments.

### Testing the resistance to UV-radiation of the cold-tolerant clones: Looking for cross-resistance

**Figure 6:**
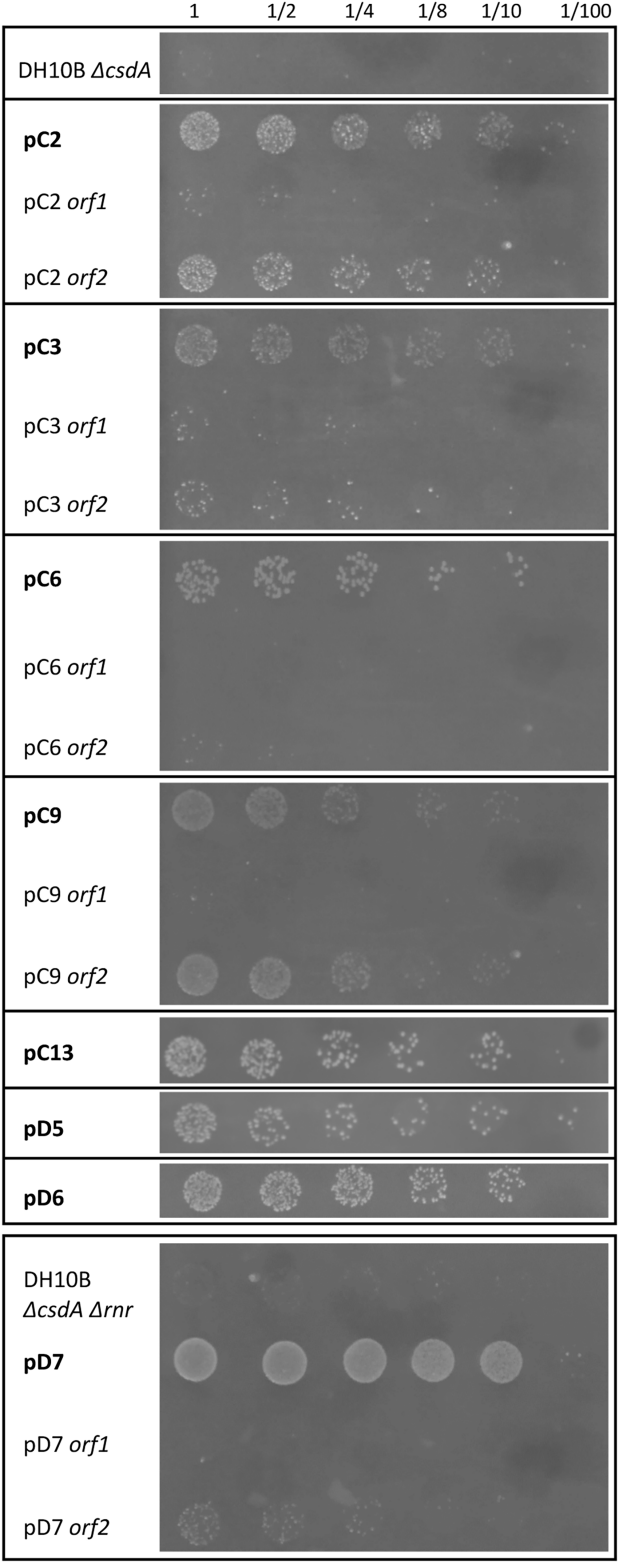
Drop assay of the cold-resistant clones and subclones that were also resistant to UV-B radiation. Both cold-sensitive strains (DH10B Δ*csdA* and DH10B Δ*csdA* Δ*rnr*) carrying empty pBluescript vector were used as negative controls. The cell density of overnight cultures was adjusted to OD_600 nm_ values of 1.0, serial dilutions were performed and 10 μl drops of each dilution were inoculated on LB-Ap_50_ plates and irradiated with a dose of UV-B of 4 mJ/cm^2^. UV-B resistance was evaluated after growing cells overnight at 37°C. Each experiment was repeated at least three times using independent cultures.

After exposing the cold-tolerant clones to UV-B radiation (4 mJ/cm^2^), we observed that 8 of the 20 clones were considerably resistant to this type of stress conditions, approximately one order of magnitude higher compared to the negative controls (DH10B *ΔcsdA* or DH10B *ΔcsdA Δrnr* strains; Figure 6). These UV-resistant clones were the following: pC2, pC3, pC6, pC9, pC13, pD5, pD6, and pD7. Of them, pC13, pD5, and pD6 are composed of a single putative ORF, thus they were considered to be responsible for the UV-resistance. pC13 encodes a hypothetical protein that contains different conserved motifs: alpha/beta hydrolase, enol-[acyl-carrier-protein] reductase (NADH) and serine aminopeptidase). pD5 encodes a hypothetical protein but, in this case, it does not have any conserved motif. Finally, pD6 encodes a GNAT family N-acetyltransferase. For the other clones that contained two ORFs (Table 1), the UV-resistance test was done with each ORF subcloned independently to know which one is directly involved in UV-resistance (Figure 6). In the case of pC2 clone, it seemed that *orf2* was clearly related to UV-resistance. It encodes for a protein that is similar (65% ID) to a 16S rRNA methyltransferase (Table 1) and that contains a conserved motif of a S-adenosyl-methionine-dependent methyltransferase. In the case of pC3 and pC6 clones, neither of their ORFs was clearly involved in UV-resistance when independently subcloned, therefore, both ORFs seemed to be needed together to protect cells from UV-radiation (Figure 6). pC9 clone also contained two ORFs and *orf2* was clearly the one involved in UV-resistance. It encodes for an unknown protein, not previously characterized, and without any known conserved motif. Finally, in the case of the pD7 clone isolated in the screening performed with the double mutant, *orf2* might be the one involved in UV-resistance but its response was much less intense than that observed for the complete clone (Figure 6). This *orf2* encodes a complete DEAD/DEAH box helicase (91% ID) and showed the typical conserved motifs of these family proteins (Table 1). The results of the control assay performed with the same clones and subclones not irradiated with UV-B radiation are compiled in Supplementary Figure S5.

## Discussion

Most of the molecular mechanisms of cold adaptation have been investigated in cultured microorganisms. Therefore, in order to expand the knowledge of these strategies, in this study, we proposed to identify new genes involved in cold adaptation from rhizosphere microorganisms of two Antarctic plants: *Colobanthus quitensis* and *Deschampsia antarctica*. To our knowledge, this is the first study applying a functional metagenomics approach (as culture-independent technique) to isolate cold-tolerance genes from environmental microorganisms. This strategy has been successfully used to study mechanisms of adaptation to diverse extreme conditions, such as acidic pH (Guazzaroni et al., 2013), metals (Mirete et al., 2007; Morgante et al., 2014), high salinity (Kapardar et al., 2010; Culligan et al., 2012, 2013; Mirete et al., 2015), UV-radiation (Lamprecht-Grandío et al., 2020), or perchlorate (Díaz-Rullo et al., 2021).

In this work, a total of 29 genes that enhanced the growth of *E. coli* cold-sensitive strains at low temperatures were identified and characterized. In this way, 21 cold-tolerant genes were isolated from *Colobanthus quitensis* library (C) and 8 from *Deschampsia antarctica* library (D). Twelve of these cold-tolerant genes (41%) were identified as genes encoding conserved hypothetical proteins (7 from library C; 1 from library D) and unknown proteins (no similarity to other proteins) (2 from library C; 2 from library D) that could be involved in new cold-tolerance mechanisms not described until now. On the other hand, 17 genes (59%) encode proteins similar to others previously characterized, including a wide variety of different potential functions that can be classified into four categories: (i) metabolic reactions (6 from library C; 1 from library D), (ii) transport and cellular processes (2 from library C), (iii) genetic information processing (3 from library C; 4 from library D); and (iv) non-totally defined function (1 from library C). Only one protein was included in the latter category: a cyclase partially encoded by the truncated gene isolated in the pC5 clone. It is difficult to identify the function of the cyclase isolated in this work, since it is truncated, and no conserved motifs have been described in it.

Of the 29 cold-tolerant genes isolated from the Antarctic metagenomic libraries, *E. coli* homologs were found for only 16 of them (10 in the specific case of the DH10B strain genome) (Table 2). The remaining 13 genes provided totally new functions for this bacterium, optimizing its growth at low temperatures. Seven of the 10 *E. coli* DH10B homologs were overexpressed in the cold-sensitive mutant DH10B *ΔcsdA* and all conferred increased cold tolerance (Figures 4, 5). In fact, all these genes have been related to cold-acclimation processes in other organisms as explained in detail below. Furthermore, some transcriptomic and proteomic analysis performed with *E. coli* cultures have confirmed an upregulation of some of them or other related genes after cold shock (Mihoub et al., 2003; Phadtare and Inouye, 2004; Vidovic et al., 2011). Therefore, these *E. coli* genes, most related to DNA repair and replication, translation, protein folding or acetyl-CoA metabolism, are involved in cold stress response and their overexpression contribute to better growth of the cold-sensitive strain in a similar way as their environmental homologs (Figures 4, 5).

### Cold-tolerant genes encoding proteins involved in metabolic reactions

Regarding the category of “metabolic reactions,” seven genes were identified that code for: pantetheine-phosphate adenylyltransferase (pC2 *orf1*), asparaginase (pC4), murein L,D-transpeptidase (pC6 *orf1*), extradiol dioxygenase (pC7), aminoacetone oxidase family FAD-binding enzyme (pC10 *orf2*), ferredoxin (pC11 *orf2*), and acyl-CoA dehydrogenase (pD1). Although these metabolic proteins have very diverse functions, many of them could be clearly connected to cold-tolerance mechanisms of adaptation. In fact, overexpression experiments performed with *E. coli* homologs for pantetheine-phosphate adenylyltransferase, asparaginase, aminoacetone oxidase, and acyl-CoA-dehydrogenase also conferred greater cold tolerance to the cold-sensitive strain (Figures 4, 5).

The pantetheine-phosphate adenylyltransferase (PPAT) and the acyl-CoA dehydrogenase (ACAD) are two proteins related to CoA metabolism. PPAT is a nucleotidyltransferase that participates in the biosynthesis of CoA from the pantothenic acid (Rubio et al., 2008) while ACAD enzyme catalyzes the first reaction of each catabolic β-oxidation cycle, favoring the release of a variable number of acetyl-CoA molecules from a fatty acid molecule until its complete dissociation (Swigonová et al., 2009). Therefore, both proteins are directly involved in increasing CoA levels, an essential cofactor in numerous biosynthetic, degradative, and energy obtaining pathways. Some authors have reported that proteins related to the biosynthesis of acetyl-CoA and ketone bodies reutilization (MetF, ScoB or MmsA) are induced at subzero temperatures in the Antarctic bacterium *Psychrobacter* sp. (Koh et al., 2017). Ting et al. (2010) detected a higher expression of the ACAD enzyme at 10°C compared to the levels detected at 30°C in the proteomic study performed with the marine bacterium *Sphingopyxis alaskensis.* Finally, acetyl-CoA is a precursor of fatty acid synthesis, and ACAD enzymes might participate in the modification of lipid membrane composition. At low temperatures, shorter and more unsaturated fatty acids conferred a greater fluidity to membranes, as it was described in the marine cyanobacteria *Synechococcus sp.* (Koh et al., 2017; Pittera et al., 2018).

As a result of the increased acetyl-CoA/CoA ratio during cold acclimation, other metabolic pathways, such as the methylglyoxal (MGO) metabolism, are also affected (Raymond-Bouchard et al., 2017). A protective role of the aminoacetone oxidase enzyme under stress conditions in the bacterium *Streptococcus oligofermentans* has been described (Molla et al., 2014). Aminoacetone is a pro-oxidative compound that can be oxidized to the toxic MGO. However, aminoacetone oxidase may protect cells by acting as an antioxidant agent, reducing cellular aminoacetone contents under stress conditions, and avoiding the production of the toxic MGO (Tressel et al., 1986; Molla et al., 2014). On the other hand, ferredoxins are electron transfer proteins that are ubiquitous in biological redox systems across all domains of life. They constitute a highly diverse family of iron–sulfur proteins that links important biochemical pathways for energy transduction, nutrient assimilation, and primary metabolism (Atkinson et al., 2016). Ferredoxins show typical characteristics of cold-adapted enzymes, such as an increased structural flexibility, a low stability at moderate temperatures, and high activity rates at low temperatures (Cvetkovska et al., 2018).

The extradiol dioxygenases constitute a large and diverse group of metalloenzymes that catalyze the rupture of aromatic rings in many different aerobic catabolic pathways (Vaillancourt et al., 2004). To our knowledge, these enzymes have not previously been related to cold adaptation in a specific way. Our results have demonstrated that this enzyme may benefit the cell growth under low temperatures since is fully linked to catabolic reactions. Moreover, extradiol dioxygenases were also related to the acetyl-CoA metabolism, which has an essential role in cold adaptation as we have previously explained (Nogales et al., 2007; Pérez-Pantoja et al., 2010).

Many amino acid metabolism reactions are altered during cold adaptation with the aim of contributing to cell survival (Sundareswaran et al., 2010; Bocian et al., 2015). The L-asparaginase is one of these enzymes whose expression is induced under low temperatures in different organisms (Phadtare and Inouye, 2004; Cho et al., 2007; Sundareswaran et al., 2010; Bocian et al., 2015). Moreover, L-asparaginase activity has been described as beneficial for cold adaptation in the psychrophilic bacteria *Pseudomonas syringae* (Sundareswaran et al., 2010). We have also isolated a cold-tolerant gene related to cell envelope metabolism that encodes a murein L,D-transpeptidase. This enzyme participates in the biosynthesis of the bacterial peptidoglycan that is part of bacterial cell walls. A thicker peptidoglycan layer and an upregulation of peptidoglycan biosynthesis have been described in different studies conducted at low temperatures (Rodrigues et al., 2008; Chen et al., 2009; Raymond-Bouchard et al., 2017; Collins and Margesin, 2019). A proteomic analysis performed with the psychrophilic bacteria *Planococcus halocryophilus* showed an increased expression of a DD-transpeptidase at −10°C of more than 9-fold compared to the values obtained at 23°C. Likewise, other enzymes related to peptidoglycan biosynthesis (MurA and MurB) also increased their expression levels at subzero temperatures (Mykytczuk et al., 2013; Raymond-Bouchard et al., 2017).

### Cold-tolerant genes encoding proteins related to transport and membrane processes

Two cold-tolerant genes possibly encoding an ABC transporter permease (pC8) and an isoprenylcysteine carboxyl methyltransferase (pC12 *orf2*) are included in the second category of “transport and cellular processes.” Both proteins may favor cellular growth under low temperatures through different mechanisms. ABC transporters participate in a wide range of physiological processes. Specifically, previous transcriptomic and proteomic analysis have demonstrated the relevant role of ABC transporters on cold adaptation due to their ability to import/export a variety of different molecules across the cytoplasmic and outer membranes that may be reused for *de novo* synthesis of different nutrients and metabolites that are essential for cold acclimation (Phadtare and Inouye, 2004; Mykytczuk et al., 2011; Koh et al., 2017). On the other hand, isoprenylcysteine carboxyl methyltransferases (ICMTs) are proteins responsible for the last step of a post-translational modification termed prenylation that can affect numerous proteins, favoring a better association of proteins to cellular membranes, protein–protein interactions, and protein stability (Zhang and Casey, 1996; Hála and Žárský, 2019). Recently, plant studies supported a possible connection between protein prenylation and its response to both abiotic and biotic stress conditions (Hála and Žárský, 2019).

### Cold-tolerant genes encoding proteins responsible for genetic information processing

Interestingly, seven cold-tolerant genes isolated in this work are included in the third category of “genetic information processing”: a 16S rRNA methyltransferase (pC2 *orf2*), a Hsp70 family protein (pC3 *orf2*), a DNA ligase (encoded by two clones: pC11 *orf1* and pD3), a leucyl-tRNA synthetase (pD4), a GNAT family N-acetyltransferase (pD6), and a DEAD/DEAH box helicase (pD7). In addition, overexpression experiments performed with *E. coli* homologs for 16S rRNA methyltransferase, Hsp70 family protein, and leucyl-tRNA synthetase also conferred higher cold tolerance to the cold-sensitive strain, which confirms its direct relation with cold acclimation (Figures 4, 5).

16S rRNA (guanine-966-N2)-methyltransferase is responsible for the methylation of the 16S rRNA molecule at the N2 position of its G966 nucleotide (Baldridge and Contreras, 2013). Nucleotide modifications are considered essential for ribosome maturation, but recent studies have also related them to response to stressful conditions such as temperature changes or antibiotic presence (Lesnyak et al., 2007; Sergiev et al., 2007). Studies performed with *E. coli* knockout mutants for methyltransferase genes have shown reduced growth rate under thermal stress compared to the wild-type strain.

Other enzymes related to RNA processing are aminoacyl-tRNA synthetases. These enzymes catalyze the binding of each amino acid to its tRNA molecule in a very specific reaction (McClain, 1993). A proteomic analysis performed with the psychrophile *Planococcus halocryophilus* showed that a higher number of proteins related to amino acid and nucleotide recycling are detected at low temperatures. In fact, a higher abundance of some tRNA synthetases (for alanine and glutamic) was detected at –10°C (Raymond-Bouchard et al., 2017). On the other hand, studies with inactive pear embryos during its dormant phase evaluated the activity of aminoacyl-tRNA synthetases and they confirmed a higher activity of these enzymes after cold treatment (Tao and Khan, 1974).

Molecular chaperones such as those of the Hsp70 family can interact with different proteins and favor their proper folding to achieve a specific functional conformation. They also participate in other cellular processes such as: molecule transport, disaggregation and degradation of different proteins, being important for many different processes related to DNA replication, cell division, and metabolism (Maillot et al., 2019). Recent studies have supported the induction of heat-shock proteins in response to low temperatures, since correct folding and refolding of cold-damaged proteins are vital after cold shock (Lelivelt and Kawula, 1995; Chow and Tung, 1998; Phadtare and Inouye, 2004; Stetina et al., 2015; Maillot et al., 2019). In fact, cell recovery rate after a freezing period at −80°C was related to the abundance of heat-shock proteins previously accumulated in *E. coli* cells (Chow and Tung, 1998).

Two cold-tolerant genes isolated in the Antarctic libraries encode DNA ligases, which are DNA-joining enzymes that are essential for survival of all organisms due to their critical roles in DNA replication and repair (Berg et al., 2019). Some genomic studies performed with *Psychrobacillus* strains have suggested an evolution of their pan-genome structures to allow their adaptation to extremely cold habitats, consisting of changing their genome contents to gain higher capacity for DNA repair, translation, and membrane transport (Choi et al., 2020). Similarly, some proteomic analyses carried out with different organisms have also supported the idea that an upregulation of proteins related to DNA repair, transcription, and translation is required to adapt to cold stress conditions (Raymond-Bouchard et al., 2017; Liu et al., 2020).

Another gene totally related to cold acclimation is the one encoding a DEAD/DEAH box helicase (pD7 *orf2*), isolated from library D in the screening performed with the double mutant DH10B *∆csdA ∆rnr*. This gene could be complementing the mutation in the *csdA* gene that encodes a highly conserved DEAD box RNA helicase essential for cold acclimation in *E. coli*, and therefore would allow the growth of this mutant strain at 15°C. DEAD box helicases such as CsdA participate in ribosome subunit biogenesis, translation initiation, cell transport, gene regulation, and stabilization/degradation of mRNAs. Alternatively, they may prevent or resolve misfolded proteins, providing assistance to rRNAs to reach their active conformation (Awano et al., 2007; Phadtare, 2012). All of these actions are essential during cold adaptation, where secondary structures of RNA are stably formed under low temperatures and helicases are required for their unwinding, for ribosome binding, and, therefore, for translation initiation (Yamanaka and Inouye, 2001; Phadtare, 2012).

Finally, clone pD6 encodes a N-terminal truncated GNAT. GNATs (general control non-repressible 5-related N-terminal acetyltransferases) constitute an important family of proteins that includes more than 10,0000 members among eukaryotes, bacteria, and archaea (Favrot et al., 2016). Acetylation appears as a major regulatory post-translational modification and is as widespread as phosphorylation. Specifically, GNAT transfers an acetyl group from acetyl-CoA to a wide diversity of substrates, from small molecules such as aminoglycoside antibiotics to macromolecules. In this way, these acetyltransferases are known to be involved in several cellular processes such as peptidoglycan recycling, detoxification pathways, production of virulence factors, iron acquisition, or redox balance (Favrot et al., 2016). Therefore, although they have not been directly connected to cold tolerance in prokaryotes, since they participate in many processes, they could also be involved in it.

### New hypothetical and unknown proteins related to cold tolerance

In addition to all these cold-tolerant genes that are similar to previously characterized genes (17 of 29 genes) and that can be connected to a cold response, some genes encoding conserved hypothetical (8) and unknown (4) proteins have also been isolated from the Antarctic metagenomic libraries. Some of these hypothetical proteins showed conserved domains that may relate them to specific functions (Table 1). In this way, the hypothetical protein encoded by the pC3 *orf1* gene might act as a spermidine synthase or as a S-adenosyl methionine-dependent methyltransferase. The hypothetical protein encoded by the pC10 *orf1* gene may be a putative ATP-dependent DNA helicase and the one encoded by the single gene of the pC13 clone might act as an alpha/beta hydrolase. For the remaining hypothetical and unknown proteins, no conserved motifs have been identified. However, some of them seem to have DNA-binding domains (predicted by both bioinformatic programs used in this work, see Materials and methods section) as the ones encoded by pC1 *orf1*, pC6 *orf2*, pC9 *orf1* or pC10 *orf1* (Table 1). Therefore, some of these proteins could be related to DNA repair or protection mechanisms or genetic information processing, both essential during cold acclimation. In conclusion, regardless of their still unknown specific function, these hypothetical or unknown proteins provide totally new functions to *E. coli* cells, favoring their growth under low temperatures and could be of interest for future research.

### Resistance to UV-B radiation of some of the cold-tolerant clones: A cross-protection against other type of stress conditions

Exposure to low temperatures produces a multitude of alterations in cells, such as decreased solute and nutrient solubility, reduced diffusion rates, increased osmotic stress, and desiccation. In addition, the formation of ice crystals can cause cell damage. An increase in solvent viscosity and gas solubility and thus higher solubility of oxygen and reactive oxygen species (ROS) are also typical in cold habitats (Collins and Margesin, 2019). In this way, continuous low temperatures are totally connected with oxidative stress conditions and cold-adapted organisms have probably also developed adaptation mechanisms for them.

According to our experiments, eight of the twenty cold-tolerant clones isolated in the Antarctic metagenomic libraries also showed a considerably resistance to UV-B radiation exposure, involving ten of the genes contained in these clones [pC2 *orf2*, pC3 (*orf1* and *orf2* acting together), pC6 (*orf1* and *orf2* acting together), pC9 *orf2*, pC13, pD5, pD6 and pD7 *orf2*] (Figure 6). Five of these genes encode hypothetical (pC3 *orf1*, pC6 *orf2*, pC13, pD5) or unknown (pC9 *orf2*) proteins with an undefined function, but in many of them, a DNA-binding domain was predicted with at least one of the two bioinformatic programs used in this work (Table 1). Therefore, they could be involved in the protection or repair of DNA damage, normally caused by UV-radiation (Lamprecht-Grandío et al., 2020). The other five cold-tolerant genes that also conferred UV-resistance are similar to previously characterized genes. In fact, four of them (pC2 *orf2*, pC3 *orf2*, pD6, and pD7 *orf2*) have been related to genetic information processing, so they could be also connected to UV protection since they encode proteins normally induced under different stress conditions, favoring translation initiation, ribosome biogenesis, gene expression regulation, stabilization/degradation of mRNA molecules, or proper protein folding (Table 1; Figure 6). Finally, the last UV-resistance gene encodes a murein L,D-transpeptidase that participates in the biosynthesis of the peptidoglycan layer, a physical barrier that can also protect cells from UV-radiation as an indirect mechanism (Liu et al., 2010).

## Conclusion

The isolation of 29 novel cold-tolerant genes in this work supports the use of a functional metagenomic approach to characterize new genes related to extreme temperature tolerance mechanisms. This work has revealed a wide variety of processes that could be related to cold-tolerance, such as different metabolic reactions, transport and membrane processes, or genetic information processing. Moreover, some of the proteins encoded by the novel cold-tolerant genes described in this work are conserved hypothetical or unknown proteins that have not previously been related to cold tolerance. Further characterization of the identified genes will improve understanding of the molecular mechanisms and metabolic pathways involved in low temperature acclimatization. On the other hand, the genes discovered in this work could be used in different biotechnological applications that require low temperatures.

## Data availability statement

The datasets presented in this study can be found in online repositories. The names of the repository/repositories and accession number(s) can be found at: https://www.ncbi.nlm.nih.gov/genbank/, MZ835316-MZ835335.

## Author contributions

PF and JG-P designed the experiments and wrote the manuscript. VM participated in the revision of the manuscript and constructed the metagenomic library. VM and JG-P collected the environmental samples. PF performed the screening, the bioinformatic analysis, and the different tests of the tolerant clones. All authors contributed to the article and approved the submitted version.

## Funding

PF was supported by a postdoctoral fellowship from the *Consejería de Educación, Juventud y Deporte, Comunidad de Madrid*, European Social Fund and Youth Employment Initiative (YEI) (PEJD-2017-POST/BIO-4333). VM was supported by a postdoctoral fellowship granted by Becas Chile 2010 Program, from the Chilean government. This research was supported by: (i) grants PGC2018-096956-B-C42, CTM2009-08648-E/ANT, and CTM2011-14777-E/ANT by the Spanish Ministry of Science and Innovation/State Agency of Research MCIN/AEI/10.13039/501100011033 and by “ERDF A way of making Europe,” and (ii) the European Commission, Horizon 2020 Framework Programme, Call: H2020-LEIT-BIO-2015-1, Project: METAFLUIDICS, GA 685474.

## Conflict of interest

The authors declare that the research was conducted in the absence of any commercial or financial relationships that could be construed as a potential conflict of interest.

## Publisher’s note

All claims expressed in this article are solely those of the authors and do not necessarily represent those of their affiliated organizations, or those of the publisher, the editors and the reviewers. Any product that may be evaluated in this article, or claim that may be made by its manufacturer, is not guaranteed or endorsed by the publisher.

## Footnotes

^1^ https://www.ncbi.nlm.nih.gov/orffinder/

^2^ https://www.ebi.ac.uk/Tools/hmmer/results/95FCB862-EF40-11E9-932A%20ACDA53F04F9B/score

^3^ http://77.68.43.135:8080/DPP-PseAAC/

^4^ https://dnabind.szialab.org/

^5^ https://browser.combase.cc/DMFit.aspx
