# Supplementary material for: Isolation of novel cold-tolerance genes from rhizosphere microorganisms of Antarctic plants by functional metagenomics
Source: Front Microbiol. 2022 Nov 18;13:1026463. doi: 10.3389/fmicb.2022.1026463 (PMC9717686; doi:10.3389/fmicb.2022.1026463)
Supplement: Supplementary file 1 [file Image_1.PDF]

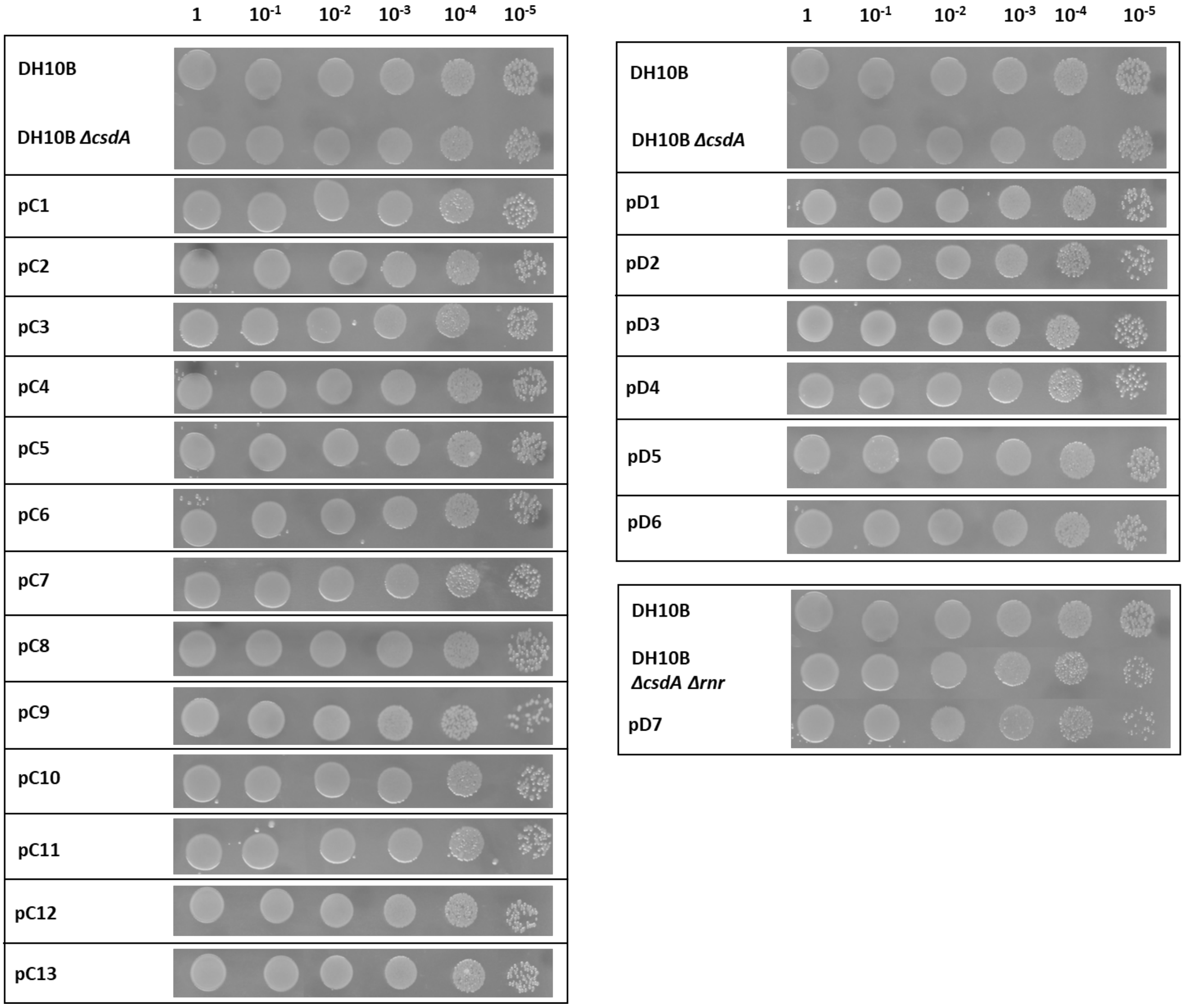

Supplementary Figure 1. Control drop assay performed with the 20 cold-resistance clones and the control strains used in the cold test (*see Fig. 1*). The cell density of overnight cultures was adjusted to $OD_{600\,nm}$ values of 1.0, serial dilutions were performed and 10 µl drops of each dilution were inoculated on LB-Ap_{50} plates. Similar cell density of the different cultures was checked growing cells overnight at 37°C.
