# Supplementary material for: Isolation of novel cold-tolerance genes from rhizosphere microorganisms of Antarctic plants by functional metagenomics
Source: Front Microbiol. 2022 Nov 18;13:1026463. doi: 10.3389/fmicb.2022.1026463 (PMC9717686; doi:10.3389/fmicb.2022.1026463)
Supplement: Supplementary file 2 [file Image_2.PDF]

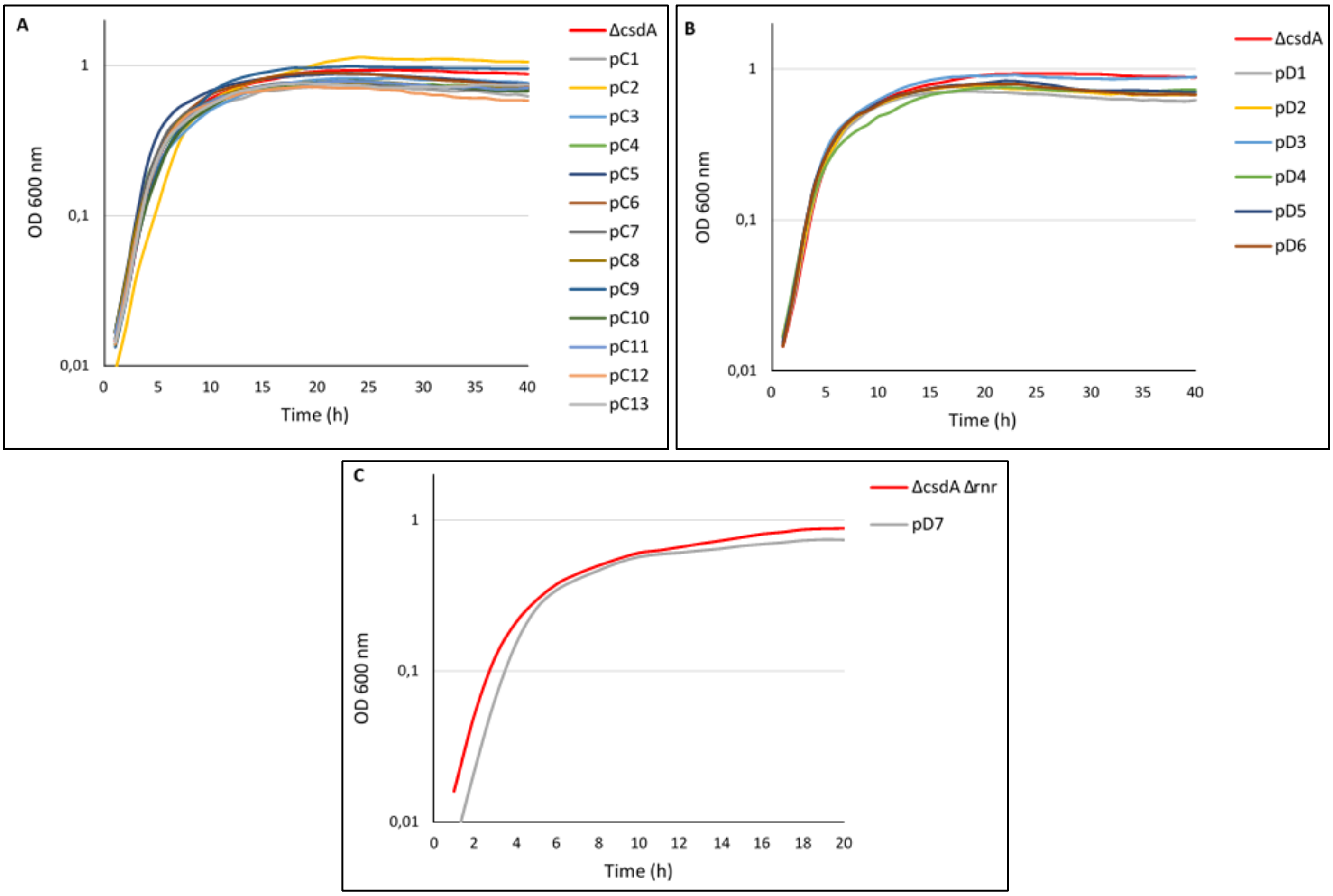

Supplementary Figure 2. Growth curves of the 20 cold-resistant clones performed at 30°C in the microplate reader TECAN Infinite M Nano during 20-40h. Both cold sensitive strains (DH10B *ΔcsdA* and DH10B *ΔcsdA Δrnr*) carrying empty pBluescript vectors were used as controls. All the clones were grown during 5h at 37°C with a slight agitation and then their $OD_{600\ nm}$ values were adjusted to 0.03. They were grown in 96-well microtiter plates during 20-40h at 30°C with a slight agitation (80 rpm). Measurements were taken automatically every hour. 4 replicates per clone were introduced in each assay and each experiment was repeated at least three times using independent cultures to corroborate the results. (**A**): Results obtained for the 13 cold-resistant clones isolated from library C (pC1-pC13) in the screening performed with the single cold-sensitive mutant (DH10B *ΔcsdA*). (**B**): Results obtained for the 6 cold resistant clones isolated from library D (pD1-pD6) in the screening performed with the single cold-sensitive mutant. (**C**): Results obtained for the cold-resistant clone pD7 isolated from library D in the screening performed with the cold-sensitive double mutant (DH10B *ΔcsdA Δrnr*).
