# Supplementary material for: Isolation of novel cold-tolerance genes from rhizosphere microorganisms of Antarctic plants by functional metagenomics
Source: Front Microbiol. 2022 Nov 18;13:1026463. doi: 10.3389/fmicb.2022.1026463 (PMC9717686; doi:10.3389/fmicb.2022.1026463)
Supplement: Supplementary file 3 [file Image_3.PDF]

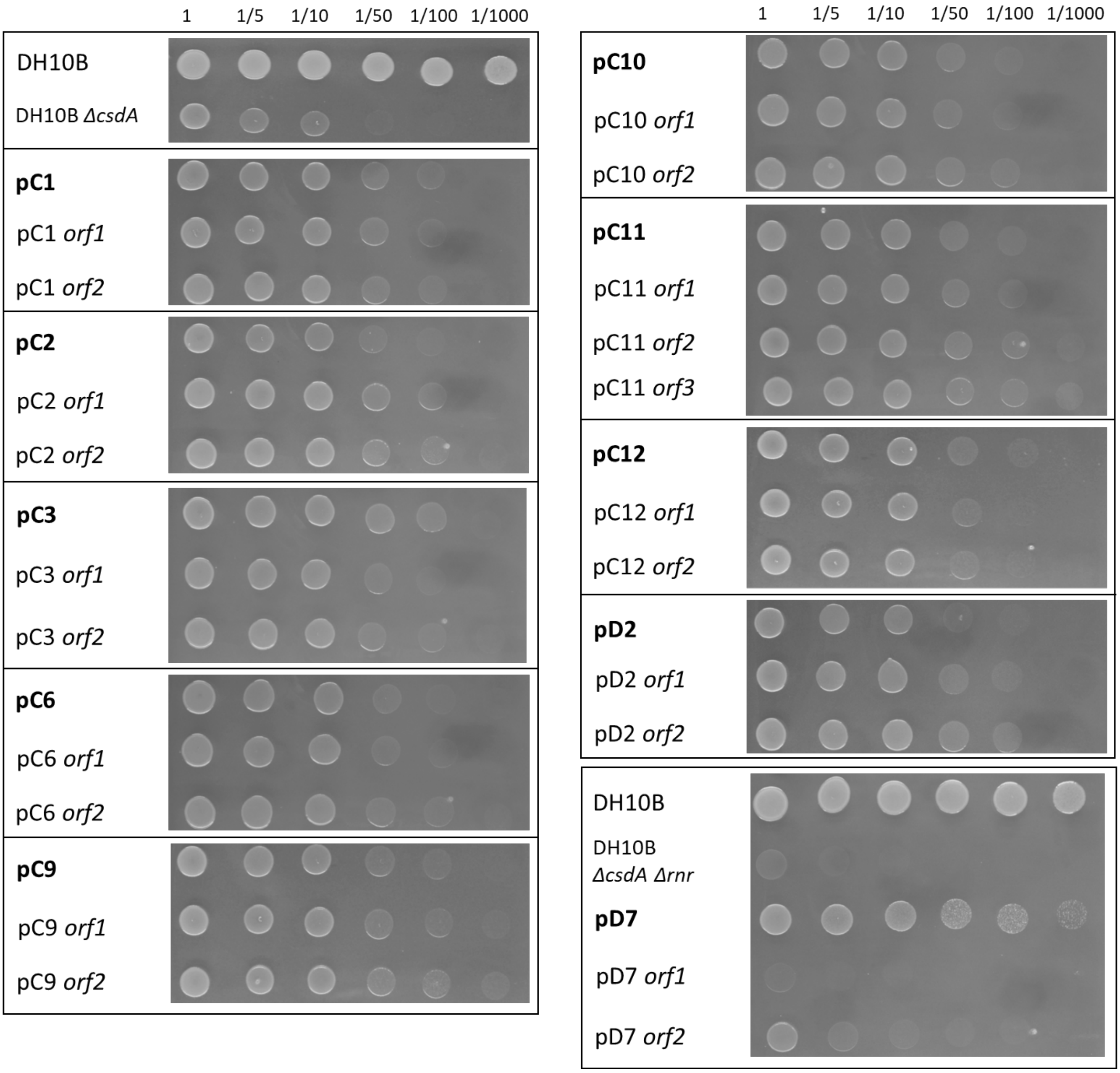

Supplementary Figure 3. Drop assay performed with the cold-resistant clones that are formed by two or more putative genes. In this cold-test, the cold-resistance of the complete clones and the subclones have been compared. DH10B strain carrying an empty pBluescript vector was used as a positive control and both cold sensitive strains (DH10B *ΔcsdA* and DH10B *ΔcsdA Δrnr*) also carrying empty pBluescript vectors were used as negative controls. The cell density of overnight cultures was adjusted to $OD_{600\ nm}$ values of 1.0, serial dilutions were performed and 10 µl drops of each dilution were inoculated on LB-Ap_{50} plates that were grown at 15°C for 10 days. Each experiment was repeated at least three times using independent cultures.
