# Supplementary material for: Isolation of novel cold-tolerance genes from rhizosphere microorganisms of Antarctic plants by functional metagenomics
Source: Front Microbiol. 2022 Nov 18;13:1026463. doi: 10.3389/fmicb.2022.1026463 (PMC9717686; doi:10.3389/fmicb.2022.1026463)
Supplement: Supplementary file 4 [file Image_4.PDF]

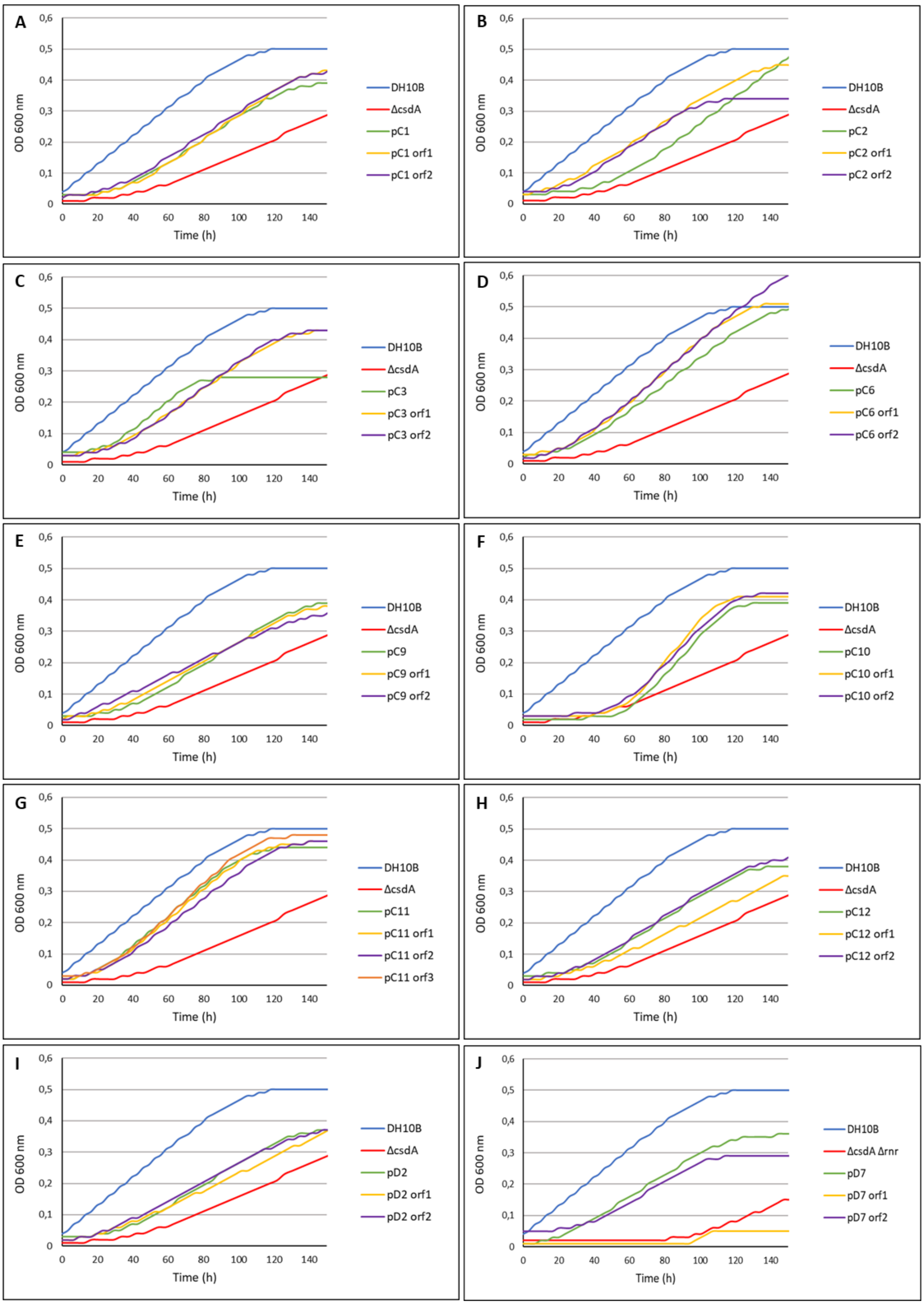

Supplementary Figure 4. Growth curves of the cold-resistant clones that are formed by two or more putative genes. In this cold-test, the cold-resistance of the complete clones and the subclones have been compared. DH10B strain carrying an empty pBluescript vector was used as a positive control and both cold sensitive strains (DH10B *ΔcsdA* and DH10B *ΔcsdA Δrnr*) also carrying empty pBluescript vectors were used as negative controls. All the clones were grown during 5h at 37°C with a slight agitation and then their $OD_{600\ nm}$ values were adjusted to 0.03. They were grown in 96-well microtiter plates during 140h at 15°C with a slight agitation (80 rpm) and OD measures were taken in the SPECTROstar Nano (BMG Labtech) four times per day. 6 replicates per clone were introduced in each assay and each experiment was repeated at least three times using independent cultures to corroborate the results.
