# Supplementary material for: Isolation of novel cold-tolerance genes from rhizosphere microorganisms of Antarctic plants by functional metagenomics
Source: Front Microbiol. 2022 Nov 18;13:1026463. doi: 10.3389/fmicb.2022.1026463 (PMC9717686; doi:10.3389/fmicb.2022.1026463)
Supplement: Supplementary file 5 [file Image_5.PDF]

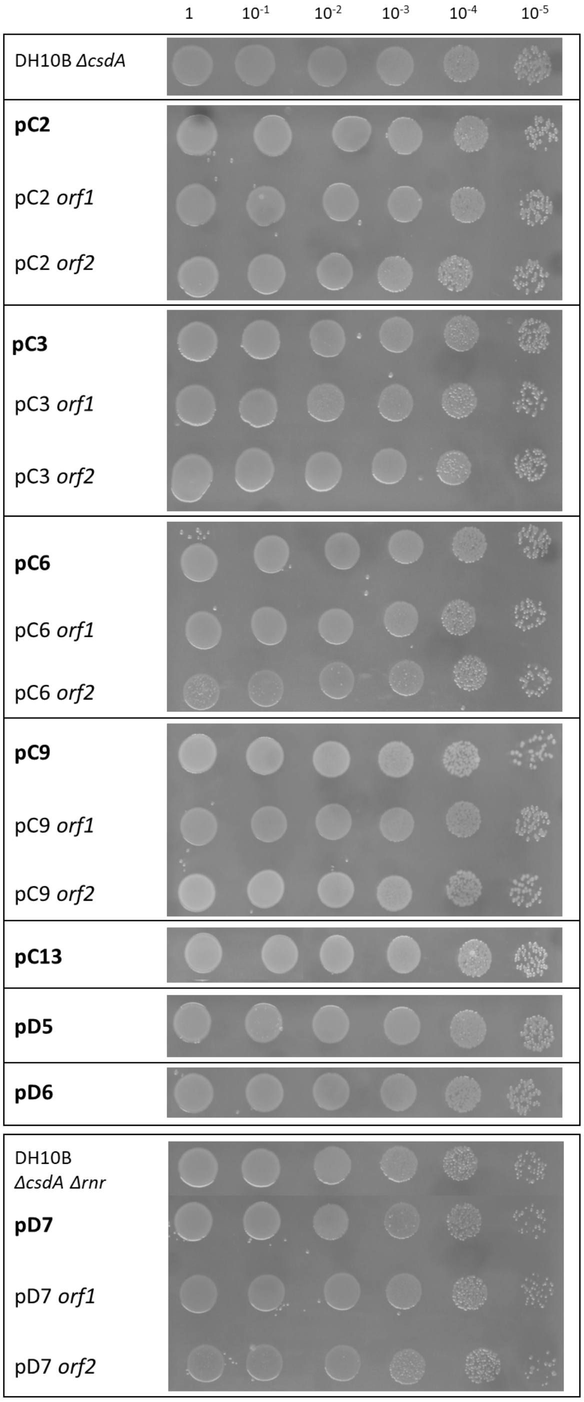

Supplementary Figure 5. Control drop assay performed with the cold-resistance clones, subclones and control strains used in the UV-B resistance test (*see Fig. 6*) but in this case they were not irradiated with UV-B radiation to check the similar cell density of the different cultures. The cell density of overnight cultures was adjusted to $OD_{600\ nm}$ values of 1.0, serial dilutions were performed and 10 µl drops of each dilution were inoculated on LB-Ap_{50} plates that were grown overnight at 37°C without being irradiated. Each experiment was repeated at least three times using independent cultures.
