# Supplementary material for: Isolation of novel cold-tolerance genes from rhizosphere microorganisms of Antarctic plants by functional metagenomics
Source: Front Microbiol. 2022 Nov 18;13:1026463. doi: 10.3389/fmicb.2022.1026463 (PMC9717686; doi:10.3389/fmicb.2022.1026463)
Supplement: Supplementary file 6 [file Table_1.PDF]

Supplementary Table 1. Primers used in this study

Primer	Sequence (5' – 3')	Use
csdA-Kn A<br>csdA-Kn B	GACGTACACATCAGCCCGTAATCT<br>GACGTTTGGGTGGTGGTTAGTG	Amplification of the DNA cassette for constructing the DH10B *ΔcsdA* strain
rnr-Cm A<br>rnr-Cm B	GCCACATTACACCTGCCTGTAG<br>CCTGATGCACTACGTTTATCCGG	Amplification of the DNA cassette for constructing the DH10B *ΔcsdA Δrnr* strain
Check csdA A<br>Check csdA B	GTTTGGTTGATGCGAGTGATTT<br>CATGACCACTGGCAGAGAAA	Confirmation of the correct insertion of the specific cassette for interrupting *csdA* gene
Check rnr A<br>Check rnr B	GATATGGGATAGTGTTCACCCTTG<br>GACTTCCTGAAAACGTTCAGGG	Confirmation of the correct insertion of the specific cassette for interrupting *rnr* gene
M13 Fw<br>M13 Rv	GTAAAACGACGGCCAGT<br>GGAAACAGCTATGACCATG	Sequencing of cold resistant clones and subcloning of independent ORFs
Subclone pC1-*orf1* Rv	CTAGTCTAGAGTCTCCTTCGGCTGC	Subcloning of the pC1 *orf1*
Subclone pC1-*orf2* Fw	CCGCTCGAGTATCATTGCCAGCCTGG	Subcloning of the pC1 *orf2*
Subclone pC2-*orf1* Rv	CTAGTCTAGATGGTCGCCGTGGAG	Subcloning of the pC2 *orf1*
Subclone pC2-*orf2* Fw	CCGCTCGAGCCTCATCGTGCAGC	Subcloning of the pC2 *orf2*
Subclone pC3-*orf1* Rv	CTAGTCTAGAACGCGTGATCCTTACC	Subcloning of the pC3 *orf1*
Subclone pC3-*orf2* Fw	CGGGGTACCCAAGGGGTGAGGGG	Subcloning of the pC3 *orf2*
Subclone pC6-*orf1* Rv	CTAGTCTAGAGCGACAAAAGGCTACG	Subcloning of the pC6 *orf1*
Subclone pC6-*orf2* Fw	CGGGGTACCCCGTAGCCTTTTGTCG	Subcloning of the pC6 *orf2*
Subclone pC9-*orf1* Rv	CTAGTCTAGACACCGCGAGGAAGG	Subcloning of the pC9 *orf1*
Subclone pC9-*orf2* Fw	CCGCTCGAGGAAGCTGAAGAAGCGC	Subcloning of the pC9 *orf2*
Subclone pC10-*orf1* Rv	CTAGTCTAGAGTCGATCAGTCTTCCGC	Subcloning of the pC10 *orf1*
Subclone pC10-*orf2* Fw	CCGCTCGAGGAAAGTGCCGCAGG	Subcloning of the pC10 *orf2*
Subclone pC11-*orf1* Rv	CTAGTCTAGACGAGAGCACCTTCTAGC	Subcloning of the pC11 *orf1*
Subclone pC11-*orf2* Fw	CCGCTCGAGCCTTCCTCGAATTCCTCC	Subcloning of the pC11 *orf2*
Subclone pC11-*orf2* Rv	CTAGTCTAGACTTTTCAAGATTGTCGTGGG	
Subclone pC11-*orf3* Fw	CGGGGTACCTAGAACTGGTTTCAAAACCG	Subcloning of the pC11 *orf3*
Subclone pC12-*orf1* Rv	CTAGTCTAGACCAACAGCCAGAGTAAGC	Subcloning of the pC12 *orf1*
Subclone pC12-*orf2* Fw	CCGCTCGAGGACGACATCTGTATTGAGC	Subcloning of the pC12 *orf2*
Subclone pD2-*orf1* Rv	CTAGTCTAGACCGAGAGATAAATTCGATTCCC	Subcloning of the pD2 *orf1*
Subclone pD2-*orf2* Fw	CCGCTCGAGGGTAGAGCGGATCTACG	Subcloning of the pD2 *orf2*
Subclone pD7 *orf1* Rv	CTAGTCTAGAAAACTACGATGCTTCCGG	Subcloning of the pD7 *orf1*
Subclone pD7 *orf2* Fw	CCGCTCGAGTACCTTGCCCATTAATCTCC	Subcloning of the pD7 *orf2*
ansB Fw<br>ansB Rv	CCGCTCGAGCACGCGGAATAATTTATTCCGG<br>CTAGTCTAGAGCAATCCTCAATCCAAACCG	Overexpression of the asparaginase gene of *E. coli* (homolog to pC4)
oxi Fw<br>oxi Rv	CCGCTCGAGCCACCTTATATTAAGCATGGAGG<br>CTAGTCTAGAGGTATGCAAATCCAGGCC	Overexpression of the aminoacetone oxidase gene of *E. coli* (homolog to pC10 *orf2*)
AcoA Fw<br>AcoA Rv	CTAGTCTAGATTCGTTATATCACTGAGCGCG<br>CCGCTCGAGTCTTGTTGGATAAATCTGGCGG	Overexpression of the acyl-CoA-dehydrogenase gene of *E. coli* (homolog to pD1)
LeuS Fw<br>LeuS Rv	CCGCTCGAGCTGAGTCCAGGATCATGACC<br>CTAGTCTAGACTTGTTCATCGTTTCATCTCCC	Overexpression of the leucyl-tRNA synthetase gene of *E. coli* (homolog to pD4)
PPAT Fw<br>PPAT Rv	CCGCTCGAGGAATTGCAGGTTTACGGGCG<br>CTAGTCTAGATTTAAAGACATTTGCGCGCGG	Overexpression of the pantetheine-phosphate adenylyltransferase gene of *E. coli* (homolog to pC2 *orf1*)
16S Met Fw<br>16S Met Rv	CCGCTCGAGAAGATATTCAGCGGGCGTGGG<br>CTAGTCTAGAGACCAAAGCCCAGCCAGG	Overexpression of the 16S rRNA methyltransferase gene of *E. coli* (homolog to pC2 *orf2*)
Hsp70 Fw<br>Hsp70 Rv	CCGCTCGAGCGATTCCGCCTTCATGCTCC<br>CTAGTCTAGACGGCGAGAAATCCCAACATCC	Overexpression of the predicted chaperone gene of *E. coli* (homolog to pC3 *orf2*)
