# Supplementary material for: Isolation of novel cold-tolerance genes from rhizosphere microorganisms of Antarctic plants by functional metagenomics
Source: Front Microbiol. 2022 Nov 18;13:1026463. doi: 10.3389/fmicb.2022.1026463 (PMC9717686; doi:10.3389/fmicb.2022.1026463)
Supplement: Supplementary file 7 [file Table_2.PDF]

Supplementary Table 2. Growth parameters defined in the liquid tests performed at 15°C for the control strains and the cold resistant clones and subclones

Clone	ORFs[a]	Growth parameters		
		Tg (h)	Growth start point (h)	Reach of OD 0.1 (h)
DH10B (C+)	-	161.20	0	13.12
ΔcsdA (C-)	-	277.25	29.40	75.60
ΔcsdA Δrnr (C-)	-	315.07	80.40	127.30
pC1	C	192.54	16.40	49.20
	(1)	187.34	22.96	52.48
	(2)	192.54	13.12	45.92
pC2	C	177.73	29.25	58.50
	(1)	192.54	9.75	35.75
	(2)	182.41	13.00	39.00
pC3	C	154.03	13.00	35.75
	(1)	177.73	16.25	42.25
	(2)	165.04	12.92	41.99
pC4	(1)	173.29	4.20	37.80
pC5	(1)	169.06	0	29.40
pC6	C	169.06	13.00	42.25
	(1)	144.41	16.25	39.00
	(2)	150.68	13.00	35.75
pC7	(1)	198.04	16.80	50.40
pC8	(1)	138.62	37.80	63.00
pC9	C	192.54	16.40	52.48
	(1)	231.05	13.12	42.64
	(2)	266.60	13.12	36.08
pC10	C	123.78	46.20	67.20
	(1)	106.63	37.80	63.00
	(2)	128.36	42.00	63.00
pC11	C	147.48	13.00	35.75
	(1)	147.48	13.00	35.75
	(2)	161.20	13.00	39.00
	(3)	133.30	16.25	35.75
pC12	C	198.04	22.96	49.20
	1	256.72	19.68	55.76
	(2)	192.54	16.40	45.92
pC13	(1)	111.80	34.56	48.96
pD1	(1)	169.06	33.60	58.80
pD2	C	203.87	16.40	52.48
	(1)	266.60	16.40	52.48
	(2)	231.05	16.40	45.92
pD3	(1)	182.41	29.40	58.80
pD4	(1)	198.04	6.70	36.85
pD5	(1)	198.04	29.40	63.00
pD6	(1)	187.34	29.40	58.80
pD7	C	210.04	13.40	40.20
	1	330.07	93.80	-
	(2)	231.05	16.75	46.90

The generation time (Tg), the moment in which cell growth starts and the moment in which an OD of 0.1 is reached are defined for each clone and subclone. Tg is calculated using the following formula: $Tg = (\ln 2)/\mu$, where μ is the specific growth rate. Each clone carrying the complete insert fragment is denoted as C (complete fragment) whereas numbers indicate individual ORFs subcloned separately. [a] Parenthesis indicate the subclones that are related to cold resistance.
